# *Streptococcus agalactiae* clones infecting humans were selected and fixed through the extensive use of tetracycline

**DOI:** 10.1038/ncomms5544

**Published:** 2014-08-04

**Authors:** Violette Da Cunha, Mark R. Davies, Pierre-Emmanuel Douarre, Isabelle Rosinski-Chupin, Immaculada Margarit, Sebastien Spinali, Tim Perkins, Pierre Lechat, Nicolas Dmytruk, Elisabeth Sauvage, Laurence Ma, Benedetta Romi, Magali Tichit, Maria-José Lopez-Sanchez, Stéphane Descorps-Declere, Erika Souche, Carmen Buchrieser, Patrick Trieu-Cuot, Ivan Moszer, Dominique Clermont, Domenico Maione, Christiane Bouchier, David J. McMillan, Julian Parkhill, John L. Telford, Gordan Dougan, Mark J. Walker, The DEVANI Consortium, Matthew T. G. Holden, Claire Poyart, Philippe Glaser

**Affiliations:** 1 Institut Pasteur, Unité de Biologie des Bacteries Pathogènes à Gram-positif, Paris 75015, France.; 2 CNRS UMR3525, Paris 75015, France.; 3 Institut Pasteur, Bioinformatics platform, Paris 75015, France.; 4 The Wellcome Trust Sanger Institute, Hinxton, Cambridge CB10 15A, UK.; 5 Australian Infectious Diseases Research Centre, School of Chemistry and Molecular Biosciences, University of Queensland, Brisbane, 4072 Queensland, Australia.; 6 Novartis Vaccines and Diagnostics, Siena 53100, Italy.; 7 Centre National de Référence des Streptocoques, Hôpitaux Universitaires, Paris Centre Cochin–Hôtel Dieu-Broca, Paris 75014, France.; 8 Institut Pasteur Genomic platform, Paris 75015, France.; 9 Institut Pasteur, Biologie des Bactéries Intracellulaires, Paris 75015, France.; 10 CNRS ERL3526, Paris 75015, France.; 11 Institut Pasteur, Collection de l’Institut Pasteur (CIP), Paris 75015, France.; 12 QIMR Berghofer Medical Research Institute, Brisbane, 7006 Queensland, Australia.; 13 Inflammation and Healing Research Cluster, University of the Sunshine Coast, Sippy Downs, 4556 Queensland, Australia.; 14 Institut Cochin, Université Sorbonne Paris Descartes, Paris 75014, France.; 15 INSERM, U1016, Paris 75014, France.

## Abstract

*Streptococcus agalactiae* (Group B *Streptococcus*, GBS) is a commensal of the digestive and genitourinary tracts of humans that emerged as the leading cause of bacterial neonatal infections in Europe and North America during the 1960s. Due to the lack of epidemiological and genomic data, the reasons for this emergence are unknown. Here we show by comparative genome analysis and phylogenetic reconstruction of 229 isolates that the rise of human GBS infections corresponds to the selection and worldwide dissemination of only a few clones. The parallel expansion of the clones is preceded by the insertion of integrative and conjugative elements conferring tetracycline resistance (TcR). Thus, we propose that the use of tetracycline from 1948 onwards led in humans to the complete replacement of a diverse GBS population by only few TcR clones particularly well adapted to their host, causing the observed emergence of GBS diseases in neonates.

*Streptococcus agalactiae*, or Group B streptococcus (GBS) generate considerable neonatal morbidity and mortality worldwide^1,2^. In the 1960s, it became the leading cause of neonatal infections in the US and in Europe. The first descriptions of the increase of GBS disease were reported in 1964 as between December 1961 to June 1963 GBS was described as the most frequent cause of neonatal sepsis at the Boston city hospital^3^ and in 1965, when Kexel and Schönbohm^4^ reported three independent cases of meningitis caused by GBS at the Children’s Clinic of the University of Bonn in Germany. However, in humans *S. agalactiae* is primarily a commensal of the digestive and urinary tracts, colonizing about 15–30% of healthy adults^1^. Surprisingly, multi-locus sequence typing (MLST) of GBS isolated from different countries showed that most human carriage and clinical isolates cluster into only five major clonal complexes (CC) (CC1, CC10, CC17, CC19 and CC23)^5^ (Table 1). Among those, CC17 strains are considered ‘hypervirulent’ as they are responsible for the vast majority of the meningitis among neonates, and for more than 80% of the late-onset disease cases appearing after 7 days of life^5,6^. In contrast, strains responsible for early-onset disease, appearing before the 6th day of life, belong to all five major CCs. A sixth CC, CC26 is common in African countries^7^. In addition to causing disease in humans, GBS is also a veterinary pathogen, causing bovine mastitis^8^. Distinct from human isolates, the majority of bovine isolates belongs to the CC67, a CC for which no human strains have been described^9^. Phylogenetic analysis of 238 isolates of human and bovine origin based on the sequences of 15 genes revealed that CC17 strains represent a homogeneous group of strains of recent origin^9^. The expansion of CC17 strains was suggested to have contributed to the emergence of GBS infections. However, until now, the events responsible for this expansion and the emergence of GBS neonatal infections were unknown.

Here we set out to examine GBS evolution and the rise of neonatal GBS infections. We sequence the genomes of 229 strains isolated between 1953 and 2011 spanning four continents. By combining phylogenetic analyses and the analysis of antibiotic resistance markers, we show that all six human associated CCs have a recent origin and that the observed homogeneity of CC17 strains results from a low rate of recombination relative to the other CCs. Most importantly, we show that the emergence of GBS diseases is associated with the replacement of the bacterial population by a limited number of tetracycline resistant (TcR) clones.

## Results

### Genome-based phylogeny reveals the expansion of a few clones

**Figure 1:**
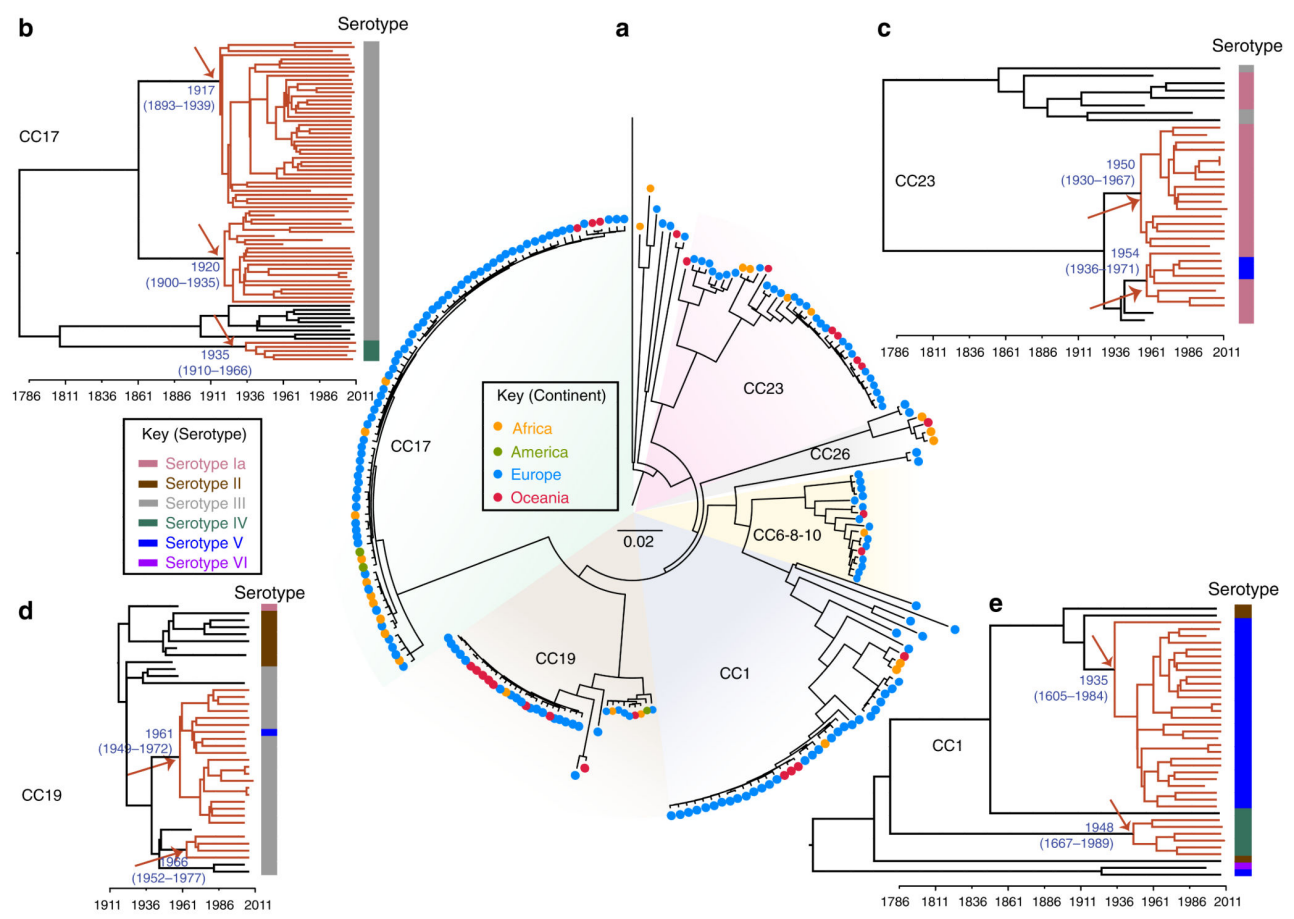
Population structure of human GBS is driven by tetracycline resistance acquisition (**a**) Whole-genome-based phylogeny of 229 sequenced GBS isolates and strain SS1219 isolated from fish^48^. Maximum Likelihood (ML) using MEGA was used to infer phylogenetic relationships. The major clonal complexes (CC) 1, 10, 17, 19, 23 and 26 as defined on the GBS MLST web site (http://pubmlst.org/sagalactiae/) correspond to well-defined branches. Isolates are indicated by dots coloured according to their geographical origin. Flanking the whole-genome phylogeny, are four Bayesian maximum clade credibility phylogenies (**b–e**) based on the non-recombinogenic genome for the GBS CC17 (**b**), CC23 (**c**), CC19 (**d**) and CC1 strains (**e**). Divergence dates (median estimates with 95% highest posterior density dates in brackets) are provided in blue for the major nodes. Coloured branches relate to the major tetracycline-resistant clones. Arrows indicate the predicted time of insertion of the ICE carrying the *tet*(M) resistance determinant within the major clones. Capsular serotypes are indicated on the right of each tree according to the indicated colour code.

The sequence of 216 GBS genomes of carriage and invasive isolates selected to represent the known diversity of the human GBS population based on MLST studies and of 13 isolates of animal origin was determined. Single-nucleotide polymorphisms (SNPs) were identified by mapping the sequence reads against the genome sequence of a representative ST19 serotype III strain, RBH11. This strain belongs to the dominant CC19 clone that has been shown to display lower levels of polymorphism with strains from other CCs^10^. In total, we identified 40,898 SNPs sites among 1,384,073 interrogated bases unambiguously mapped in the 229 genomes. Using maximum likelihood phylogenetic methods on SNPs in the core chromosome, we determined that 97% of the 216 human isolates clustered into six well-resolved lineages that correspond to the six major human CCs (Fig. 1a) as defined by MLST (Table 1). The average polymorphism between lineages is 0.52% (ranging from 0.36 to 0.65%). Strikingly, we observed that within each CC, one or two dominant clones showing limited polymorphisms are present, representing 83% of all isolates. Such a signature is indicative of an evolutionary bottleneck, which has resulted in a recent reduction in population size and expansion of positively selected clones.

### GBS CCs except for CC17 show a high rate of recombination

**Figure 2:**
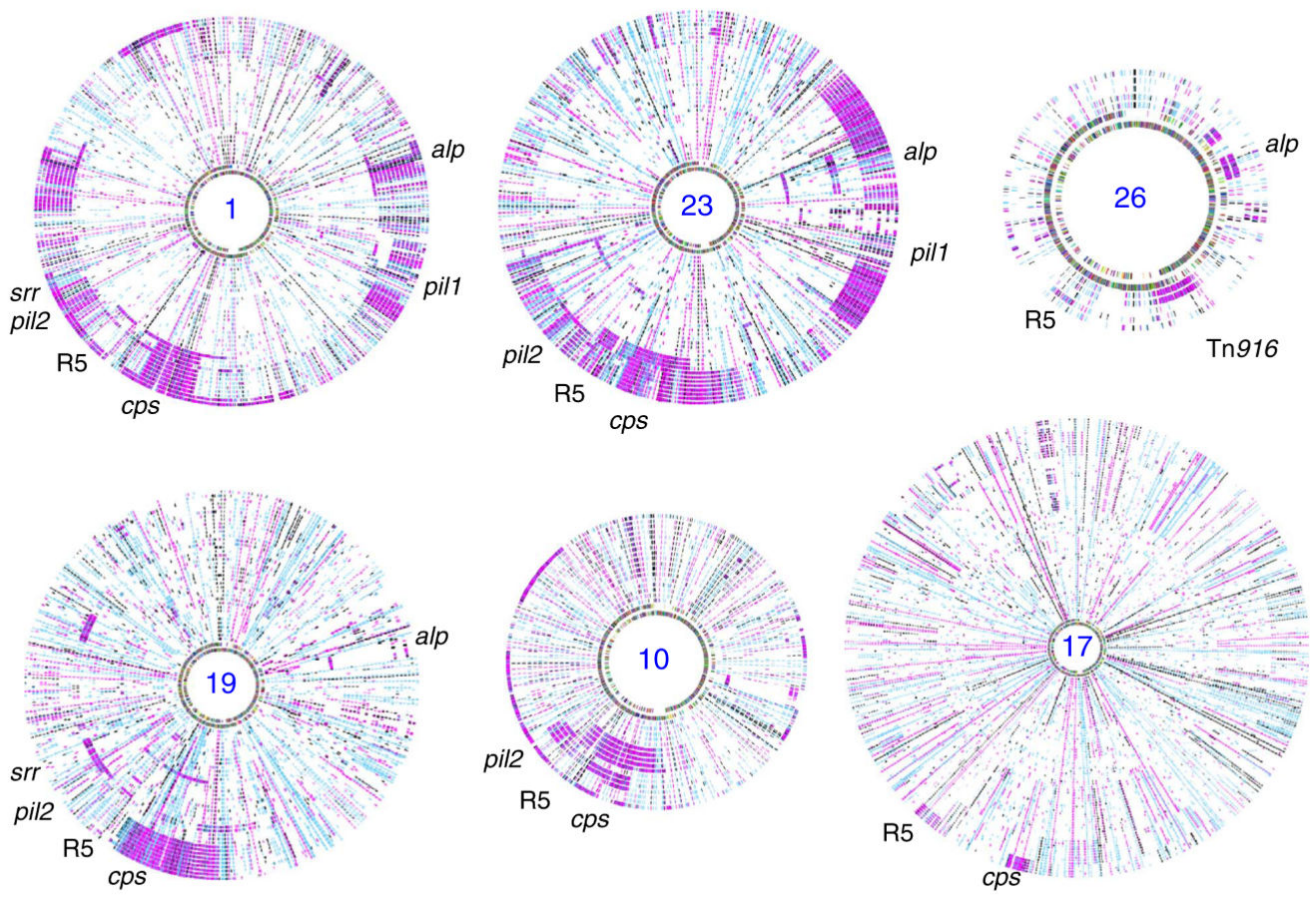
Distribution of SNPs and recombination across all GBS isolates from the six major CCs The maps were generated by using the SyntView software. Isolates were ordered according to the distance from the reference genome depicted at the inner circle. CC numbers are indicated in the centre. Recombined regions compared with the reference genome correspond to regions with a higher density of SNPs indicated by short lines on each circle corresponding to one strain. Around the outside circle are the relative positions of selected antigenic loci. The reference genomes were BG-NI-011 for CC1, DK-NI-008 for CC10, COH1 for CC17, RBH11 for CC19, CCH210801006 for CC23 and Bangui-IP-105 for CC26.

Based on the comparison of eight GBS genome sequences, we previously showed that the recombination of large chromosomal segments is a major contributor to the genomic diversification of GBS^10^. Indeed, based on the SNP distribution, here we identified evidence of recombination events encompassing all parts of the core genome (Supplementary Fig. 1), thus distorting the true phylogenetic relationships between GBS lineages. Recombination within each single CC was extensive involving 47% of the genome in CC1, 44% in CC10, 24% in CC19, 42% in CC23 and 19% in CC26 strains but was surprisingly low in CC17, with only 3% (Fig. 2 and Table 2). Most recombination events included genetic determinants for known surface antigens, such as genes encoding the capsular polysaccharide biosynthesis proteins, the major antigenic protein Rib/Alp, the R5 protein, the serine-rich protein and the pili (Table 2), which modulate host–cell interactions^11^. It is therefore likely that immune pressure is a major driving force in the genome diversification of human GBS.

### Specific GBS clones expanded in the mid-20th century

**Figure 3:**
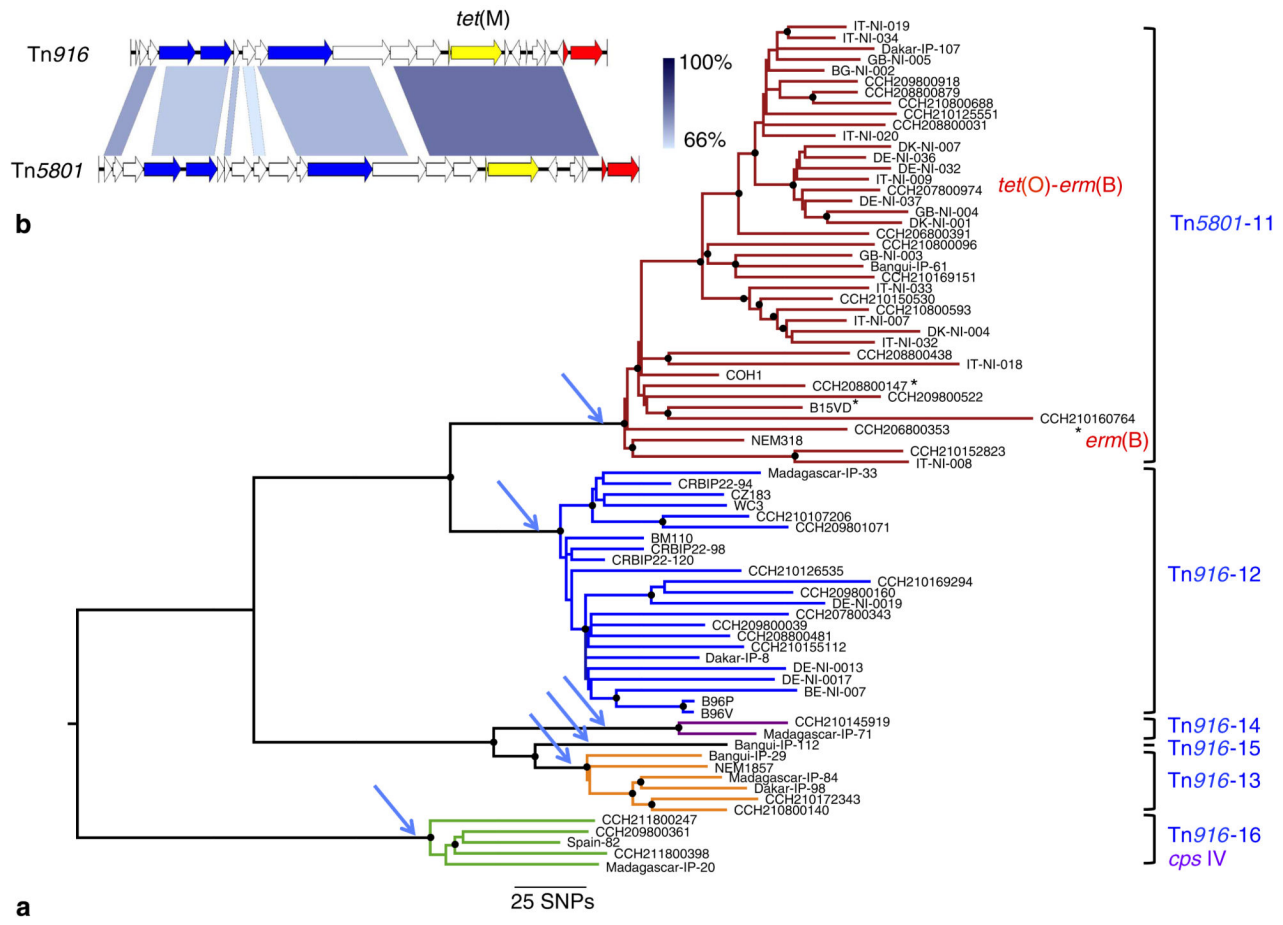
Phylogeny of the ‘hypervirulent’ CC17 lineage (**a**) ML phylogeny based on the alignment of 3,922 polymorphic positions. Six independent ICE insertions (indicated on the right and by blue arrows) corresponding to six different lineages (indicated by different colours) were identified and are numbered from 11 to 16 (Table 4). A star indicates that Tn*5801* has been lost by this isolate. Following the loss of Tn*5801*, strains CCH210160764 and CCH207800974 have acquired unrelated ICE expressing *tet*(M) and *erm*(B), and *tet*(O) and *erm*(B), respectively. Nodes with >90% bootstrap support are indicated by black dots. (**b**) Genetic maps and alignment of Tn*916* and Tn*5801*. Comparisons were performed by BLASTn. The *tet*(M) gene is coloured in yellow, genes encoding type 4 secretion system components are in blue and the integrase and excisionase genes which are not conserved between the two transposons in red. Percentages of identities are shown in blue scale and range between 68 and 98% for the *tet*(M) region.

**Figure 4:**
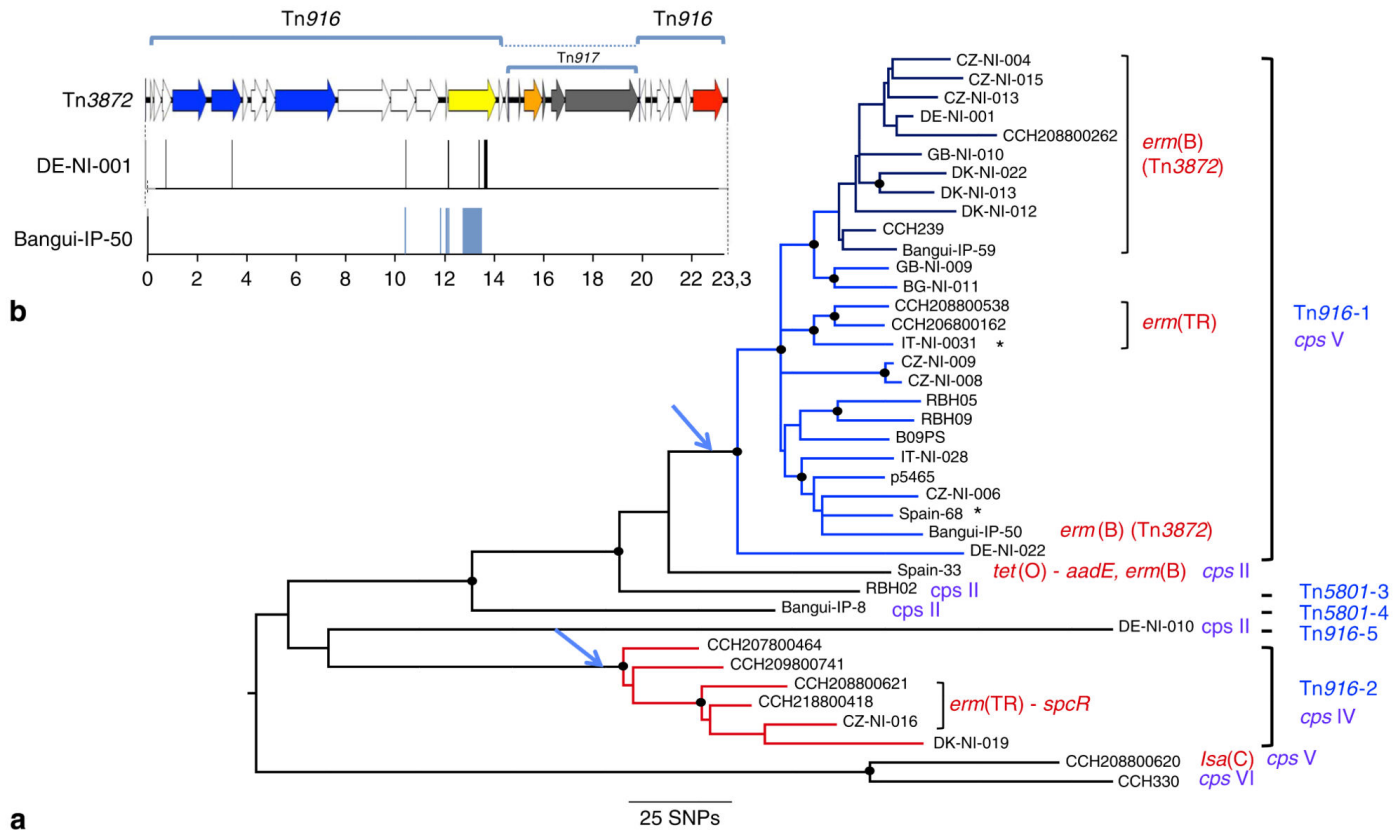
Phylogeny of clonal complex CC1 (**a**) ML phylogeny from the alignment of pseudosequences of the 1,244 polymorphic positions in 914 interrogated kbases. The five independent Tn*916* or Tn*5801* insertions are indicated in blue and numbers from 1 to 5 refer to their description in Table 4. The two TcR lineages with more than one isolate are coloured in blue and red. Three sub-lineages have acquired an *erm* resistance gene. Within lineage Tn*916*-1, 40% of the isolates (12) carry Tn*3872* (dark-blue branch and strain Bangui-IP-30). The four observed serotypes (*cps*) II, IV, V and VI are indicated in violet. A star indicates that Tn*916* has been lost by the isolate. Antibiotic resistance genes other than *tet*(M) are indicated in red. Nodes with >90% bootstrap support are indicated by black dots. (**b**) Genetic map of Tn*3872*. Tn*917* carrying the *erm*(B) gene is in grey the *erm*(B) gene being in orange, Tn*916* genes are coloured as in Fig. 3. The location of 13 out of the 15 SNPs between strain CZ-NI-006 Tn*916* and strain DE-NI-001 Tn*3872*, indicated by black bars are located between positions 10,407 and 13,659 (Tn*916* coordinates) and the 76 SNPs between strain Bangui-IP-50 and strain CZ-NI-006, all located between position 10,407 and 13,497 are in blue.

**Figure 5:**
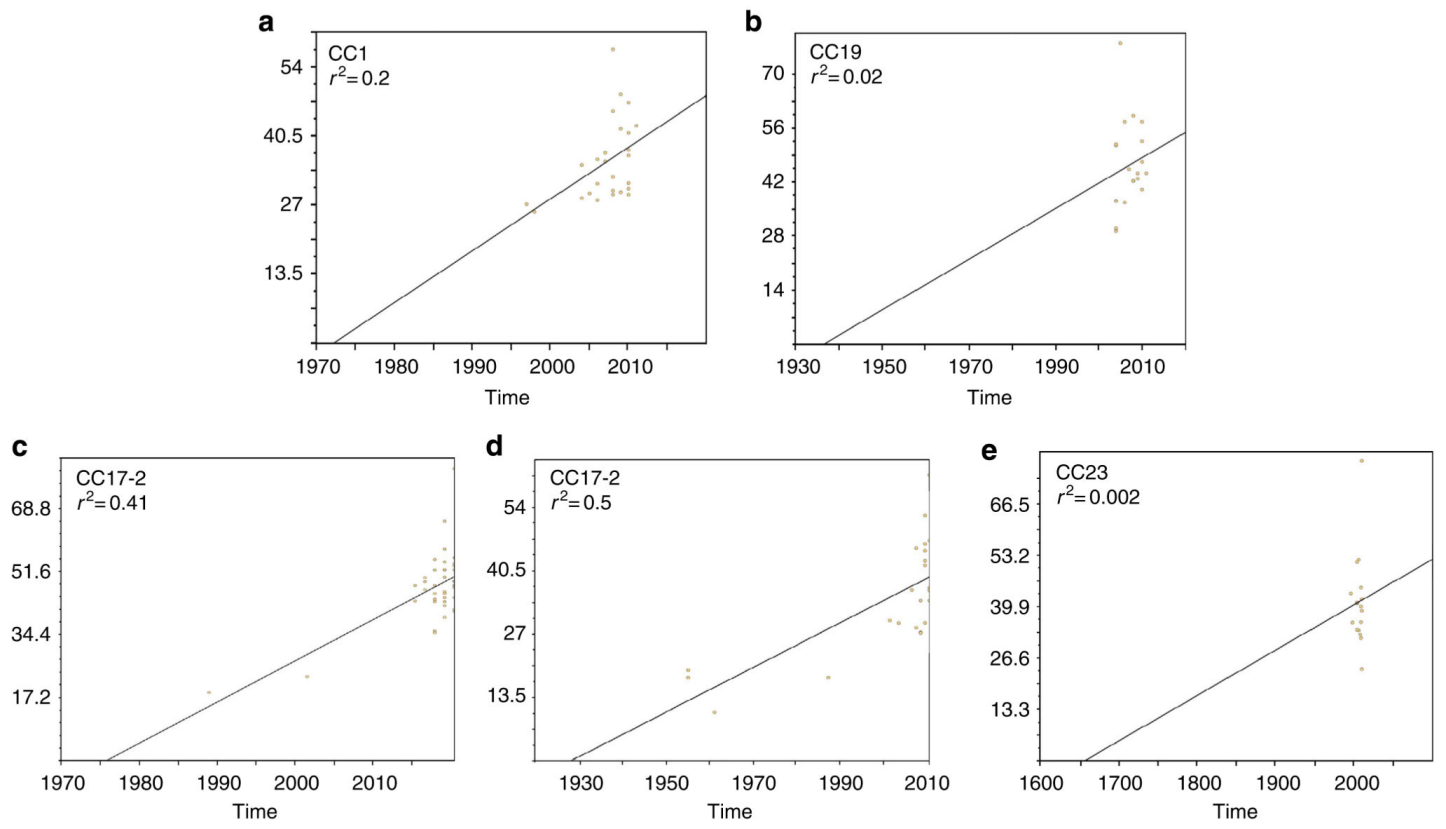
Correlation of isolation date with maximum likelihood root-to-tip branch length for the five major TcR lineages calculated with Path-O-Gen These analyses predict the origin of these clones in agreement with the BEAST analysis except for the CC23 lineage where there was a lack of temporal sampling to support tree root estimates. *X* axis, time in years; *Y* axis root-to-tip branch length in SNP per Mb. (**a**) CC1 lineage Tn*916*-1; (**b**) CC19 lineage Tn*916*-17; (**c**) CC17 lineage Tn*5801*-11; (**d**) CC17 lineage Tn*916*-12; (**e**) CC23 lineage Tn*5801*-23.

To non-ambiguously reconstruct the evolutionary history of the six GBS lineages, we performed evolutionary studies separately for each CC after mapping the sequencing reads against a representative strain of the CC and filtering out the recombined regions (Figs 3 and 4, Supplementary Figs 2–4, and Table 2). This showed that strains of each of the six CCs acquired only a very small number of SNPs, since their respective last common ancestor ranging from 74 to 174 SNPs per Mb. To date the last common ancestor of these clusters and of the nodes corresponding to the major sublineages, we performed temporal Bayesian analyses (BEAST)^12^ of CC1, CC17, CC19 and CC23 for which the number of isolates and the timespan of isolations provided sufficient posterior support (Fig. 1b–e). This analysis estimated that these CCs emerged from a common ancestor within the last 300 years, at a mutation rate of 0.56–0.93 SNP per Mb per year (Fig. 1b–e; Table 2). These values are in-between the mutation rate predicted for the hypervirulent 027/BI/NAP1 *Clostridium difficile* variant (0.15–0.53 mutations per year per Mb)^13^ and for the globally expanded multidrug-resistant PMEN1 pneumococcus (1.57 mutations per year per Mb)^14^. Nevertheless, the diversification of the six CCs predates by several decades the clinical emergence of GBS neonatal infections that took place in the 1960s^3^; therefore, the increased incidence of neonatal infections cannot be directly connected to the selection of these clones. Conversely, the majority of the predominant subclones within CC1, CC17, CC19 and CC23, were predicted to have emerged between 1917 and 1966 (Fig. 1b–e), a time period corresponding to the observed emergence of neonatal GBS infections. Linear regression of maximum likelihood root-to-tip distances against the year of sampling showed a strong correlation with this data (Fig. 5). This temporal analysis indicates that an evolutionary bottleneck has played a major role in altering the population structure of human GBS within the mid-20th century.

### Human GBS isolates belong mainly to a small number of TcR clones

A striking feature of human GBS strains is their high rate (>90%) of TcR, which is predominantly linked with the *tet*(M) gene encoding an elongation factor G-like protein. Among recent GBS studies, *tet*(M) was found in 95% of human isolates in Romania^15^, 85% in Kuwait^16^ and 91% in Tunisia^17^. In contrast, *tet*(M)-associated TcR is much less frequent among strains of bovine origin. Specifically, 58% of animal GBS isolates from Portugal were TcR with only 22% expressing *tet*(M)^18^. Similar observations have been made in France where 39% are TcR and 21% express *tet*(M)^19^, Estonia, where 22% of strains exhibited varying levels of TcR^20^ and in GBS strains isolated from milk in the state of New York where only 14.5% were TcR and 2.5% expressed *tet*(M)^21^. Furthermore, the *tet*(M) gene was detected in only 25% of the 51 bovine GBS genome sequences and 10% of the genome of 22 GBS strains isolated from fish that were available at the NCBI database as of 15 May, 2014. These values highlight a very high incidence of *tet*(M)-associated TcR within human isolates compared with animal GBS. It is known that the use of antibiotics results in a strong positive selection for antibiotic-resistant clones and an overall reduction in bacterial population diversity^22^. To identify a possible link between the population structure of human GBS and antibiotic resistance, we analysed the resistome of the 229 sequenced genomes (Table 3). In agreement with the epidemiological surveys, the most frequent determinants in the 216 human isolates were *tet*(M) (*n* = 183, 84%) and *tet*(O) (*n* = 10, 5%) both encoding ribosomal protection proteins, and *erm*(B) (*n* = 18, 9%) and *erm*(TR) (*n* = 12, 5.5%) encoding methylases conferring resistance to macrolides and lincosamides. Other resistance determinants were rare (Table 3). Except for one strain, *tet*(M) was carried by two related integrative and conjugative elements (ICEs): Tn*916* (*n* = 111) or Tn*5801* (*n* = 70) (Fig. 3b). In the collection sequenced, Tn*916* and Tn*5801* were found in 23 and 2 different insertion sites, respectively (Table 4 and Supplementary Table 1). Importantly, the expanded clones in all six CCs represent monophyletic lineages sharing the TcR-conferring ICE inserted at the same position (Table 4; Supplementary Table 1; Figs 3a and 4a and Supplementary Figs 2–4). Moreover, for a given TcR lineage, each transposon shows a maximum of five SNPs supporting a single insertion event for each lineage. These data strongly suggest that the acquisition of ICE harbouring *tet*(M) has been a landmark event in the expansion of these lineages and in the emergence of the human pathogenic GBS population. Tetracycline was first used in 1948 therefore the subsequent expansion of TcR GBS clones is in agreement with our temporal analysis dating the origin of these clones in the mid-20th century (Fig. 1b–e). Interestingly, the major clones include strains from different European and African countries and from Australia, indicating that following their selection, these clones spread globally.

GBS genomes contain a large number of ICEs and other integrative elements. To determine whether the acquisition of other genomic islands has also contributed to the expansion of these clones, we systematically searched for genes specifically acquired by the four major TcR lineages to which the reference genomes belong (CC1 lineage Tn*916*-1, CC17 Tn*5801*-11, CC19 Tn*916*-17 and CC23 Tn*5801*-23). However, we did not find evidence based on the sequenced genomes that such events have taken place.

### Macrolide resistance contributed to CC1 expansion

Tetracycline is not used to treat GBS infections; the most commonly employed antibiotics are ß-lactams, followed by macrolides^23^. Several epidemiological studies reported an increase in the incidence of macrolide resistance in GBS, in particular in strains belonging to the CC1 (ref. 24). Indeed, among the 32 isolates of our collection that contained an *erm* gene, 19 belonged to CC1 (50% of the sequenced CC1 collection) whereas the remaining 13 were sporadically distributed among the other CCs (Table 3). Among the 19 *erm*-positive CC1 isolates, 17 clustered into three lineages nested into TcR lineages (Fig. 4a). In the largest of these three lineages (*n* = 11 isolates), the *erm*(B) gene is carried by Tn*917* inserted into Tn*916* leading to Tn*3872* first described in *S. pneumoniae*^25^. A twelfth isolate, Bangui-IP-50, which belongs to another sub-lineage of the CC1 Tn*916*-1 lineage, also carries a Tn*3872* element. Comparison of the ancestral Tn*916* and the two versions of Tn*3872* showed that the majority of the SNPs (12 out of 15, strain DE-NI-001 and the 76 SNPs, strain Bangui-IP-50) clusters upstream of Tn*917* (Fig. 4b). This observation suggests that Tn*917* was inserted in Tn*916* by recombination following the conjugative transfer of two versions of Tn*3872*; however, we cannot rule out an integration of Tn*3872* following the loss of Tn*916*. In *S. agalactiae*, Tn*3872* was first identified in four serotype V isolates and one serotype Ia isolate showing similar macro restriction patterns^26^. The *erm*(B) sequences in these strains are identical to the one present in the major macrolide resistance sub-lineage we have identified, but shows three SNPs over 738 bp in comparison with the Bangui-IP-50 *erm*(B) gene. Furthermore, the insertion site reported for Tn*3872* is the same as the insertion site of Tn*916*-1. It is therefore likely that the four serotype V isolates carrying Tn*3872* previously described belong to this sub-lineage. These findings show that the *erm* genes were gained after the acquisition of Tn*916*. Therefore, acquisition of macrolide and lincosamide resistance genes occurred after the selection of the TcR clones, and in the case of the ST1 Tn*916*-1 lineage, has contributed to its clonal expansion.

## Discussion

Our analyses indicate that the observed emergence of GBS infections in the 1960s was not due to past under-diagnosis and case reporting of the disease^27^ but that it corresponds to a dramatic modification of the human GBS population. Indeed, we show that the human disease-causing and carriage GBS population is dominated be few TcR clones that have spread globally. Tetracycline is a broad-spectrum antibiotic, which was first used clinically in 1948. Subsequently it became widely used, with the first reported case of TcR described in *Shigella dysenteriae* in Japan in 1953 (ref. 28). This timeframe is compatible with the Bayesian estimates that predicted the origin of the GBS TcR clones in four CCs in the mid-20th century (Fig. 1b–e) and the identification of three TcR isolates dating back to 1955 within the CC17 clone carrying Tn*916*-12. These three isolates represent the oldest known strains carrying Tn*916* (ref. 29). Strikingly, unlike non-CC17 isolates, and despite the larger number of sequenced isolates (*n* = 79), all CC17 strains belong to clones that have acquired Tn*916* or Tn*5801* (Fig. 3a and Table 4). The absence of a CC17 population naive for these ICEs suggests that CC17 strains were rare before the evolutionary bottleneck exerted by the widespread use of tetracycline. Thus, our data allow us to propose that the acquisition of TcR led to the expansion of hypervirulent CC17 clones that then contributed to the increase of neonatal infections.

Due to the broad distribution of resistance in many bacterial species, the use of tetracycline in human health has declined^30^. However, in contrast to what is usually observed for other bacterial species when antibiotic selection pressure decreases, TcR among human GBS remains remarkably stable at the same very high level (>90%). These observations suggest that once inserted in the GBS genome, Tn*916* and Tn*5801* are maintained, as they seem to impose a limited fitness cost to GBS. Indeed, it has been suggested that due to the intricate mechanism of Tn*916* regulation, Tn*916* gene expression likely results in minimizing the biological cost to the host^31,32^. It should be noted that the dominance of the *tet*(M) gene carried by these ICEs is not specific to GBS^31^. Indeed, 133 of 271 *Enterococcus faecalis* genome sequences available in GenBank as of 15 May, 2014 contain a *tet*(M) gene, while only 11 contain *tet*(S) and one *tet*(O). Furthermore, TcR genes remain the most abundant antibiotic resistance genes identified in human gut microbiota as recently reported^33^. Therefore, the strong selective pressure that tetracycline usage has exerted is more widely observed, but in the case of GBS it has led to the emergence of pathogenic clones.

Recombination through the conjugative transfer of large genomic regions has a major role in the diversification of GBS lineages (Fig. 2). In particular, serotype switching due to recombination of the chromosomal region encompassing the capsular biosynthesis locus is the event the most frequently observed. This is also supported by overlaying the capsular serotype distribution onto the Bayesian phylogeny (Fig. 1b–e). Indeed, the majority of strains within CC1, CC17, CC19 and CC23 cluster into a dominant capsular serotype (*cps*): CC1/*cps*V, CC17/*cps*III, CC19/*cps*III and CC23/*cps*Ia. However, there is a still substantial heterogeneity within CCs relating to capsular serotype. In the case of CC19 and CC23, capsular switching is evident within the major TcR clone. Like the other major CCs examined in this study, isolates belonging to CC17 share a recent origin, however, they differ from the other CCs by a low rate of recombination. Indeed, in this CC, we detected only two recombination events in the early branch leading to the serotype IV ST256 sub-lineage (Table 2). No recombination was identified among CC17 strains, although we have previously shown that, under laboratory conditions, these strains are as permissive to conjugative transfers and recombination as non-CC17 strains^10^. Furthermore, all CC17 strains encode a specific repertoire of surface proteins shown to contribute to increased adherence to intestinal, choroid plexus and microvascular endothelial cells^6^. These observations suggest that CC17 strains colonize a specific habitat leading to genetic isolation.

In GBS, the vast majority of Tn*916* and Tn*5801* are inserted in the core genome (Table 4). Therefore, these ICE were acquired by conjugation and *tet*(M) is likely the major adaptive function not directly associated with genes encoding virulence factors as it has been described in enterococci^34^. However, the expansion of these TcR clones does not explain the concurrent emergence of neonatal infections. We propose as a model that among the selected TcR clones, those with a higher potency to colonize and to disseminate have been selected and have spread across continents replacing less virulent clones. The higher rate of TcR strains and more particularly of the *tet*(M) gene in human compared with bovine isolates is in support of this hypothesis. A link between antibiotic resistance and increased virulence or transmissibility has been observed for different lineages of opportunistic pathogens leading to the concept of ‘superbugs’^35^. Examples are the *C. difficile* clone 027/BI/NAP1, among which two highly virulent lineages expanded following the independent acquisition of resistance to fluoroquinolones^13^, and the epidemic community-acquired methicillin-resistant *Staphylococcus aureus* clone USA300, which is more virulent than hospital acquired MRSAs, and which emerged from CC8 *S. aureus* following the acquisition of the methicillin resistance-encoding staphylococcal cassette chromosome *mec* (SCC*mec*) type IV element, and the arginine catabolite mobile element^36^. In USA300, the type I arginine catabolite mobile element is integrated proximal to the SCC*mec* element, and carries a *speG* gene which encodes a spermidine acetyl transferase that has been shown to contribute to the dissemination and to the virulence of these strains^37^. Another striking example is the *Enterococcus faecium* hospital-associated lineages ST17, ST18 and ST78 that are all virulent and resistant to multiple antibiotics including vancomycin^38,39^. *E. faecium* hospital-adapted lineages show a distinct genetic repertoire compared with carriage strains and phylogenomic analysis indicated that they are unrelated to human carrier isolates but they originate from animal strains^39^. However, unique to GBS, the recent evolutionary bottleneck due to the use of tetracycline led to the global replacement of the GBS population colonizing humans. In particular, this bottleneck led to the fixation of the CC17 lineage, which shows a higher virulence in neonates^6^. Therefore, our data strongly suggest that the widespread use of tetracycline has helped drive the emergence of GBS neonatal infections, and created pandemic drug-resistant clones which pose a continued threat to human health.

## Methods

### *Streptococcus agalactiae* (GBS) isolates

GBS strains isolated between 1953 and 2011 are listed in Supplementary Table 1. Their geographical origin, the epidemiological status (early-onset disease; late-onset disease; adult invasive disease, AInv; neonatal invasive disease, NInv; carriage or of animal origin), the year of isolation, the capsular serotype (*cps*) and the sequence type are indicated. Strains were predominantly isolated from Europe (nine countries), Africa (three countries) and Australia. Carriage and invasive strains were selected to reflect the distribution of isolates according to MLST studies (Table 1). CC17 strains were over represented in the collection in an attempt to help characterize the specificity of this hypervirulent lineage. The 12 strains isolated between 1953 and 1961 from the Institut Pasteur bacterial strain collection (CRBIP) belong to CC17, CC19 and CC23. The earliest isolates from the CC1 lineage are from 1997, therefore contributing to lower confidence in dating the origin of this particular lineage (Fig. 1e). Thirteen strains are of animal origin.

### Sequencing, mapping and SNP detection

Genomes were sequenced using the Illumina GAIIX or HiSeq2000 sequencing platforms. The read length for each genome is specified in Supplementary Table 1. The minimal coverage was 75-fold. The sequencing reads were filtered to exclude reads with a quality score of <25 in 10% of the reads and aligned to the reference sequence by using Burrows-Wheeler Aligner^40^. SNP calling was done using SAMtools MPILEUP and varFilter^41^. We excluded SNPs identified in repeated regions, within 100 bp of the contig boundaries from draft reference genome assemblies, or in regions with coverage lower than 40% of the strain’s mean coverage. Reference genomes selected as belonging to the dominant clone of each CC are described in Table 2. The genome of strain COH1 was completely finished by closing the gaps of the previously determined draft genome sequence^42^. Finishing was performed by Sanger sequencing of PCR products corresponding to gaps and to low-quality regions. The COH1 genome was annotated as previously described^43^. Briefly, coding sequences were defined by combining Genmark predictions with visual inspection of each open-reading frame for the presence of a start codon with an upstream ribosome-binding site and BLASTp similarity searches on Uniref 90, Trembl and Swissprot databases. Function predictions were based on blastp similarity searches and on the analysis of motifs using the Pfam databases (http://pfam.xfam.org/). The five other reference genomes were obtained after ordering the Velvet assembled contigs by using the MAUVE software^44^ and the genome of 2603V/R as reference^45^. They were automatically annotated by using the RAST server^46^.

### *De novo* assembly and sequence analysis

Genome sequences were assembled using the Velvet software^47^ with an optimized *k*-value and a minimal coverage of 10. MLST types were determined by extracting the sequences of the seven genes of the GBS MLST system^5^ and comparing them with the known STs from the GBS MLST web server (http://pubmlst.org/sagalactiae/). Serotypes were determined by BLASTn similarity search using as query the nucleotide sequences of the ten *cps* loci corresponding to the 10 known GBS serotypes. Antibiotic resistance genes were searched by BLASTx search with the protein sequences of 38 resistance genes from Gram-positive bacteria for tetracycline, macrolides, streptomycin, kanamycin, spectinomycin, streptothrycin, lincosamides and chloramphenicol. Genomic islands encoding antibiotic resistance genes were analysed by extracting the sequences surrounding the antibiotic resistance genes. Tn*916* and Tn*5801* insertion sites were determined by analysing the chromosome–transposon junctions (Table 4).

### Phylogenetic analysis

A phylogeny of the 229 GBS isolates sequenced here and the fish isolate SS1219 (ref. 48) was constructed by considering nucleotides corresponding to all variable positions located in the core genome for example, in regions present in all isolates. Individual phylogenies of the six CCs were performed after removal of the genomic islands not shared by all isolates, and recombined regions. Automatic detection of recombined regions using the Gubbins software^14^ was used to predict genome variation that had arisen by homologous recombination. The ability of this method to robustly predict recombination across the whole population was limited due to the observed genetic diversity of the population, and the corresponding deep branches of the phylogeny. We therefore combined this automatic detection with the visual inspection of variation in each of individual CCs in SyntView^49^ viewer (http://genopole.pasteur.fr/SynTView/flash/Streptococcus_agalactiae), to identify clusters of SNPs likely to have arisen by recombination. Phylogenetic relationships were inferred using the Maximum Likelihood (ML) method in MEGA5 version 5.10 (ref. 50) and the BEAST software v1.7.5 (ref. 51). The confidence of the ML tree was estimated using the bootstrap method with 200 bootstrap replications. The trees of the six individual CCs were rooted by using the sequence of an isolate belonging to another CC. The species tree is unrooted. The Bayesian phylogenetic software of BEAST v1.7.5 (ref. 12) was used to investigate the temporal evolution of CC1, CC17, CC19 and CC23. The non-recombined variable sites as defined above for each CC was used as markers of ancestral polymorphisms and the year of isolation for each isolate (Supplementary Table 1) was used to calibrate the clock rate. We used the GTR model of nucleotide substitution with four discrete gamma-distributed rate categories and a default gamma prior distribution of 1. To identify the most suitable models, we compared the strict, lognormal relaxed and exponential-relaxed molecular clock models and coalescent constant, exponential growth, expansion growth and Bayesian skyline tree models, using stepping-stone sampling and comparison of log marginal likelihoods^52–54^. Model analyses were conducted in triplicate for 50 million Markov Chain Monte Carlo generations with samples taken every 1,000 generations. Replicate analyses were combined with 10% removed as burn-in using LogCombiner^12^. The Bayesian skyline tree model along with the uncorrelated lognormal relaxed molecular clock to accommodate for rate variation among lineages was preferred. Therefore, final phylogenetic analyses were run in triplicate for 100 million Markov Chain Monte Carlo generations with 10% burn-in using the above parameters. Overall congruency of Bayesian models were observed for all four CCs analysed by BEAST; however, the lack of a wide temporal sampling of strains for CC1 resulted in the lack of an accurate root to accurately define a clock rate, hence the broad confidence values observed in Fig. 1e. Path-O-Gen (http://tree.bio.ed.ac.uk/software/pathogen/) was used to identify a ‘molecular clock’ evolutionary signal as defined by a linear association between the ML root-to-tip branch lengths and year of strain isolation for the five major TcR sub-lineages. This also enabled an alternative method to the Bayesian approach of BEAST to date the expansion event of the five major TcR sub-lineages. ML trees were generated in the sub-lineages: CC1 Tn*916*-1 (*n* = 26); CC19 Tn*916*-17 (*n* = 20); CC17 Tn*5801*-11 (*n* = 41); CC17 Tn*916*-12 (*n* = 23) and CC23 Tn*5801*-2 (*n* = 18) from the same recombination-removed SNP alignments used for BEAST analyses.

## Supplementary Material

Supplementary data

## Figures and Tables

**Table 1:** Summary of global multi-locus sequence typing studies*.

Study†	Origin‡	Clinical origin§	Number of isolates	CC1 (ST1)∥	CC10¶	CC17	CC19 (ST19, 28)∥	CC23	CC26	Other
Jones^5^	World	Ca, Ninv, Ainv	152	16% (14%)	18%	30%	17% (13%–2%)	12%	2%	5%
Luan^55^	Sweden (1988–1997)	Ninv, Ainv	158	15% (9%)	13%	24%	29% (16%–1%)	14%	0	4%
Manning^56^	Canada Alberta (1993–2002)	Ca, Ninv	413	23% (19%)	14%	16%	20% (19%–NA)	22%	NA	5%
Bohnsack^57^	USA (1995–1999)	Ca, Ninv	899	16% (15%)	9%	13%	17% (12%–2%)	40%	0	5%
Sadowi^58^	Poland (1996–2005)	Ca, Ninv, Ainv	114	17% (13%)	18%	14%	12% (9–2%)	37%	1%	1%
Huber^59^	Kenya (2007–2010)	Ca, Ainv	169	12% (9%)	17%	21%	14% (5%–4%)	27%	2%	7%
Brochet^7^	Dakar Bangui (2006–2007)	Ca	163	20% (9%)	6%	12%	28% (4%–15%)	17%	15%	2%

NA, Not available.

* As percentage in each clonal complex.

† First author of the publication and reference.

‡ In parenthesis the years of isolation if known.

§ The epidemiological origin: Ca, carriage, Ninv, neonatal invasive disease, Ainv, adults invasive disease.

∥ In parenthesis, the percentage corresponding to the indicated sequence types (STs).

¶ Corresponds to strains from the closely related clonal complexes (CC) CC6, CC8 and CC10.

**Table 2:** Diversity within the six clonal complexes.

	CC1	CC10	CC17	CC19	CC23	CC26
Strain name	BG-NI-011	DK-NI-008	COH1	RBH11	CCH210801006	Bangui-IP-105
Genome size*	2,078 (25)	2,080 (34)	2,065 (1)	2,180 (34)	2,055 (17)	2,054 (45)
No. of isolates	39	18	79	39	36	6
Interrogated regions†	914 kb	1,069 kb	1,860 kb	1,427 kb	980 kb	1,582 kb
Polymorphic positions	1,244	971	3,922	2,016	1,329	263
Recombination‡	987 kb *pil1, pil2, cps*, R5, *alp, srr*	914 kb *pil1, pil2, cps*, R5, *alp, srr*	68 kb *cps*, R5	532 kb *cps*, R5, *pil2,* *srr, alp*	875 kb *pil1, pil2, cps*, R5, *alp, srr*	398 kb Tn*916*/CRISPR, R5, *alp*
Depth§	174±23	126±7	129±16	97±21	169±16	74±34
Mutation rate∥	0.64	—	0.56	0.93	0.75	—

* Total contig size in kb, in parenthesis, the number of contigs.

† Cumulative size in kb of regions not predicted to have recombined.

‡ Cumulative size in kb of the recombined regions and the exchanged antigenic loci.

§ Expressed as the average number of single-nucleotide polymorphism (SNP) per Mb from the root to the tips of the tree for strains isolated after 2005.

∥ As estimated by dividing the depth of each lineage by its age predicted by the BEAST analysis, in SNPs per Mb per year (Fig. 1b–e).

**Table 3:** Distribution of antibiotic resistance genes among the 229 sequenced isolates.

Antibiotic resistance	Gene	Number of isolates							Total
		CC1	CC10	CC17	CC19	CC23	CC26	Other	
Tetracycline	*tet*(M)	34	13	77	27	27	5	7	190
	*tet*(O)	1	2	1	6	1	0	0	11
	*tet*(L)	0	1	0	0	1	2	0	4
Erythromycin	*erm*(B)	13	0	2	4	0	0	0	19
	*erm*(TR)	6	0	0	2	2	2	0	12
	*erm*(T)	0	1	0	0	0	0	0	1
	*mefE*	0	0	0	0	1	0	0	1
Streptomycin	*aadE*	1	0	0	4	1	0	0	6
Kanamycin	*aph3’*-III	0	0	0	3	1	0	0	4
Streptothrycin	*sta*	0	0	0	3	1	0	0	4
Chloramphenicol	*cat*	0	0	0	2	0	0	0	2
Lincosamides	*lnuB*	0	0	0	1	0	0	0	1
Lincosamide—streptogramin	*lsa*(C)	1	1	0	2	1	0	1	6
Spectinomycin	*spcR*	3	0	0	2	1	2	0	8
Total number of isolates		39	19	78	39	36	6	12	229

**Table 4:** Characteristics of clones deriving from Tn*916* and Tn*5801* insertions.

Clone				No. of isolates per clone	Integrative and conjugative element and site* of insertion	Recombination events within the clone†
*N*	CC	Sequence type	*cps*			
1	1	1	V	27 (2)‡	Tn*916* GI 624 kb§,,∥	92 kb *alp*; 100 kb
2		196, 459	IV	6	Tn*916* GI 624 kb§,,∥	80 kb *alp*: 35kb
3		2	IV	1	Tn*5801* 972 kb¶	— #
4		2	IV	1	Tn*5801* 972 kb¶	n.a.
5		2	II	1	Tn*916* 726 kb	n.a.
				3**		n.a.
6	10	10	V-II	5	Tn*5801* 972 kb¶	347 kb *cps* operon
7		8	Ib	4	Tn*916* 846 kb	—
8		12	II	1	Tn*916* 260 kb	n.a.
9		10	Ib	1	Tn*916* 626 kb	n.a.
10		10	II	2	Tn*916* 1,255 kb	—
				5**		n.a.
11	17	17	III	42 (3)‡	Tn*5801* 972 kb¶	—
12		17	III	23	Tn*916* 241 kb	—
13		17	III	6	Tn*916* 950 kb	—
14		17	III	2	Tn*916* 1,928 kb	—
15		17	III	1	Tn*916* 1,928 kb	n.a.
16		291	IV	5	Tn*5801* 52 kb	—
17	19	19	III V	20 (3)‡	Tn*916* 923 kb	253 kb *cps* operon 36 kb R5 protein
18		19	III	4 (1)‡	Tn*916* GI 557 kb∥	—
19		19	III	1	Tn*916* 2,005 kb	n.a.
20		28	II	2	Tn*916* 395 kb	—
21		28	II	3	Tn*916* 1,445 kb	—
22		28	II	1	Tn*916* 2,005 kb	n.a.
				8**		n.a.
23	23	23	Ia	18 (1)‡	Tn*5801* 972 kb¶	107 kb *alp*; 56 kb R5 protein; 93 kb
24		23, 144	Ia–V	8 (1)‡	Tn*916* 1,928 kb	91 kb *alp*; 193 kb *cps*
25		23	III	1	Tn*5801* 972 kb¶	n.a.
26		23	Ia	1	Tn*5801* 972 kb¶	n.a.
				8**		n.a.
27	26	26	V	3	Tn*916* GI 1,023 kb∥	—
28		26	V	2	Tn*916* 923 kb	77 kb R5 protein
29	22	22	II	2	Tn*916* 1787 kb	—

n.a., Not applicable.

* Position relative to the completely sequenced 2603 V/R genome^45^.

† Size of the recombined regions and antigenic loci.

‡ In parenthesis are indicated the number of strains predicted to have lost Tn*916* or Tn*5801*.

§ Tn*916* is inserted in the opposite orientation at the same site.

∥ Tn*916* is inserted within genomic islands inserted at the reported locations relative to the 2603 V/R genome.

¶ 972 kb correspond to a hot spot of insertion of Tn*5801* at the 5′ end of the *guaA* gene.

# A dash indicates no recombination event.

** Number of isolates in clonal complex (CC)1, 10, 19 and 23 predicted not to have acquired Tn*916* or Tn*5801*.

## Author contributions

V.D.C., M.R.D. and M.T.G.H. analysed data and wrote the manuscript; P.-E.D. and I.R.-C. performed experiments and analysed data; I.Ma., T.P., E.S., L.M., B.R., M.T., M.-J.L.-S., P.T.-C., D.M., C.Bo. and J.P. performed experiments; S.S. and N.D. provided GBS isolates; P.L., S.D.-D., E.S. and I.Mo. provided software and bioinformatics expertise; C.Bu. and M.J.W. wrote the manuscript; J.L.T., G.D. and M.J.W. designed the experiments, The DEVANI members, D.C. and D.J.M. selected and provided GBS isolates; C.P. selected and provided GBS isolates and wrote the manuscript, P.G. conceived the experiments, selected isolates, analysed data and wrote the manuscript.

## References

[1] Edwards MS, Baker CJ, Klein JO, Remington JS (2005). Group B streptococcal infections. Infectious Diseases of the Fetus and Newborn Infant.

[2] Edmond KM (2012). Group B streptococcal disease in infants aged younger than 3 months: systematic review and meta-analysis. Lancet.

[3] Eickhoff TC, Klein JO, Daly AK, Ingall D, Finland M (1964). Neonatal sepsis and other infections due to group B beta-hemolytic streptococci. N. Engl. J. Med.

[4] Kexel G, Schoenbohm S (1965). *Streptococcus agalactiae* as the causative agent in infantile meningitis. Dtsch. Med. Wochenschr.

[5] Jones N (2003). Multilocus sequence typing system for group B streptococcus. J. Clin. Microbiol.

[6] Tazi A (2010). The surface protein HvgA mediates group B streptococcus hypervirulence and meningeal tropism in neonates. J. Exp. Med.

[7] Brochet M, Couve E, Bercion R, Sire JM, Glaser P (2009). Population structure of human isolates of *Streptococcus agalactiae* from Dakar and Bangui. J. Clin. Microbiol.

[8] Nocard M, Mollereau H (1887). Sur unemammite contagieuse des vaches laitieres. Ann. Inst. Pasteur.

[9] Sorensen UB, Poulsen K, Ghezzo C, Margarit I, Kilian M (2010). Emergence and global dissemination of host-specific *Streptococcus agalactiae* clones. MBio.

[10] Brochet M (2008). Shaping a bacterial genome by large chromosomal replacements, the evolutionary history of *Streptococcus agalactiae*. Proc. Natl Acad. Sci. USA.

[11] Lindahl G, Stalhammar-Carlemalm M, Areschoug T (2005). Surface proteins of *Streptococcus agalactiae* and related proteins in other bacterial pathogens. Clin. Microbiol. Rev.

[12] Drummond AJ, Suchard MA, Xie D, Rambaut A (2012). Bayesian phylogenetics with BEAUti and the BEAST 1.7. Mol. Biol. Evol.

[13] He M (2012). Emergence and global spread of epidemic healthcare-associated *Clostridium difficile*. Nat. Genet.

[14] Croucher NJ (2011). Rapid pneumococcal evolution in response to clinical interventions. Science.

[15] Usein CR (2012). Molecular characterization of adult-colonizing *Streptococcus agalactiae* from an area-based surveillance study in Romania. Eur. J. Clin. Microbiol. Infect. Dis.

[16] Boswihi SS, Udo EE, Al-Sweih N (2012). Serotypes and antibiotic resistance in Group B *streptococcus* isolated from patients at the Maternity Hospital, Kuwait. J. Med. Microbiol.

[17] Hraoui M, Boutiba-Ben Boubaker I, Rachdi M, Slim A, Ben Redjeb S (2012). Macrolide and tetracycline resistance in clinical strains of *Streptococcus agalactiae* isolated in Tunisia. J. Med. Microbiol.

[18] Rato MG (2013). Antimicrobial resistance and molecular epidemiology of streptococci from bovine mastitis. Vet. Microbiol.

[19] Haenni M (2010). Diversity and mobility of integrative and conjugative elements in bovine isolates of *Streptococcus agalactiae, S. dysgalactiae* subsp. *dysgalactiae*, and *S. uberis*. Appl. Environ. Microbiol.

[20] Kalmus P, Aasmae B, Karssin A, Orro T, Kask K (2011). Udder pathogens and their resistance to antimicrobial agents in dairy cows in Estonia. Acta. Vet. Scand.

[21] Dogan B, Schukken YH, Santisteban C, Boor KJ (2005). Distribution of serotypes and antimicrobial resistance genes among *Streptococcus agalactiae* isolates from bovine and human hosts. J. Clin. Microbiol.

[22] Baquero F, Tedim AP, Coque TM (2013). Antibiotic resistance shaping multi-level population biology of bacteria. Front. Microbiol.

[23] Verani JR, McGee L, Schrag SJ (2010). Prevention of perinatal group B streptococcal disease–revised guidelines from CDC, 2010. MMWR Recomm. Rep.

[24] De Francesco MA, Caracciolo S, Gargiulo F, Manca N (2012). Phenotypes, genotypes, serotypes and molecular epidemiology of erythromycin-resistant *Streptococcus agalactiae* in Italy. Eur. J. Clin. Microbiol. Infect. Dis.

[25] McDougal LK (1998). Detection of Tn*917*-like sequences within a Tn*916*-like conjugative transposon (Tn*3872*) in erythromycin-resistant isolates of *Streptococcus pneumoniae*. Antimicrob. Agents Chemother.

[26] Puopolo KM, Klinzing DC, Lin MP, Yesucevitz DL, Cieslewicz MJ (2007). A composite transposon associated with erythromycin and clindamycin resistance in group B streptococcus. J. Med. Microbiol.

[27] Patterson MJ, El Batool Hafeez A (1976). Group B streptococci in human disease. Bacteriol. Rev.

[28] Chopra I, Roberts M (2001). Tetracycline antibiotics: mode of action, applications, molecular biology, and epidemiology of bacterial resistance. Microbiol. Mol. Biol. Rev.

[29] Wyres KL (2013). Evidence of antimicrobial resistance-conferring genetic elements among pneumococci isolated prior to 1974. BMC Genomics.

[30] Thaker M, Spanogiannopoulos P, Wright GD (2010). The tetracycline resistome. Cell. Mol. Life. Sci.

[31] Roberts AP, Mullany P (2011). Tn916-like genetic elements: a diverse group of modular mobile elements conferring antibiotic resistance. FEMS Microbiol. Rev.

[32] Celli J, Trieu-Cuot P (1998). Circularization of Tn*916* is required for expression of the transposon-encoded transfer functions: characterization of long tetracycline-inducible transcripts reading through the attachment site. Mol. Microbiol.

[33] Hu Y (2013). Metagenome-wide analysis of antibiotic resistance genes in a large cohort of human gut microbiota. Nat. Commun.

[34] Arias CA, Murray BE (2012). The rise of the *Enterococcus*: beyond vancomycin resistance. Nat. Rev. Microbiol.

[35] Davies J, Davies D (2010). Origins and evolution of antibiotic resistance. Microbiol. Mol. Biol. Rev.

[36] Tenover FC, Goering RV (2009). Methicillin-resistant *Staphylococcus aureus* strain USA300: origin and epidemiology. J. Antimicrob. Chemother.

[37] Planet PJ (2013). Emergence of the epidemic methicillin-resistant *Staphylococcus aureus* strain USA300 coincides with horizontal transfer of the arginine catabolic mobile element and *speG*-mediated adaptations for survival on skin. MBio.

[38] Willems RJ (2012). Restricted gene flow among hospital subpopulations of *Enterococcus faecium*. MBio.

[39] Lebreton F (2013). Emergence of epidemic multidrug-resistant *Enterococcus faecium* from animal and commensal strains. MBio.

[40] Li H, Durbin R (2009). Fast and accurate short read alignment with Burrows-Wheeler transform. Bioinformatics.

[41] Li H (2009). The Sequence Alignment/Map format and SAMtools. Bioinformatics.

[42] Tettelin H (2005). Genome analysis of multiple pathogenic isolates of *Streptococcus agalactiae*: implications for the microbial ‘pan-genome’. Proc. Natl Acad. Sci. USA.

[43] Glaser P (2002). Genome sequence of *Streptococcus agalactiae*, a pathogen causing invasive neonatal disease. Mol. Microbiol.

[44] Darling AC, Mau B, Blattner FR, Perna NT (2004). Mauve: multiple alignment of conserved genomic sequence with rearrangements. Genome Res.

[45] Tettelin H (2002). Complete genome sequence and comparative genomic analysis of an emerging human pathogen, serotype V *Streptococcus agalactiae*. Proc. Natl Acad. Sci. USA.

[46] Aziz RK (2008). The RAST Server: rapid annotations using subsystems technology. BMC Genomics.

[47] Zerbino DR, Birney E (2008). Velvet: algorithms for *de novo* short read assembly using de Bruijn graphs. Genome Res.

[48] Rosinski-Chupin I (2013). Reductive evolution in *Streptococcus agalactiae* and the emergence of a host adapted lineage. BMC Genomics.

[49] Lechat P, Souche E, Moszer I (2013). SynTView—an interactive multi-view genome browser for next-generation comparative microorganism genomics. BMC Bioinformatics.

[50] Tamura K (2011). MEGA5: molecular evolutionary genetics analysis using maximum likelihood, evolutionary distance, and maximum parsimony methods. Mol. Biol. Evol.

[51] Drummond AJ, Rambaut A (2007). BEAST: Bayesian evolutionary analysis by sampling trees. BMC Evol. Biol.

[52] Baele G (2012). Improving the accuracy of demographic and molecular clock model comparison while accommodating phylogenetic uncertainty. Mol. Biol. Evol.

[53] Baele G, Lemey P (2013). Bayesian evolutionary model testing in the phylogenomics era: matching model complexity with computational efficiency. Bioinformatics.

[54] Drummond AJ, Ho SY, Phillips MJ, Rambaut A (2006). Relaxed phylogenetics and dating with confidence. PLoS Biol.

[55] Luan SL (2005). Multilocus sequence typing of Swedish invasive group B streptococcus isolates indicates a neonatally associated genetic lineage and capsule switching. J. Clin. Microbiol.

[56] Manning SD (2009). Multilocus sequence types associated with neonatal group B streptococcal sepsis and meningitis in Canada. J. Clin. Microbiol.

[57] Bohnsack JF (2008). Population structure of invasive and colonizing strains of *Streptococcus agalactiae* from neonates of six U.S. Academic Centers from 1995 to 1999. J. Clin. Microbiol.

[58] Sadowy E, Matynia B, Hryniewicz W (2010). Population structure, virulence factors and resistance determinants of invasive, non-invasive and colonizing *Streptococcus agalactiae* in Poland. J. Antimicrob. Chemother.

[59] Huber CA, McOdimba F, Pflueger V, Daubenberger CA, Revathi G (2011). Characterization of invasive and colonizing isolates of *Streptococcus agalactiae* in East African adults. J. Clin. Microbiol.

