# Supplementary material for: Streptococcus agalactiae clones infecting humans were selected and fixed through the extensive use of tetracycline
Source: Nat Commun. Author manuscript; Available in PMC 2015 Aug 17. (PMC4538795; doi:10.1038/ncomms5544)
Supplement: Supplementary data [file NIHMS64295-supplement-Supplementary_data.pdf]

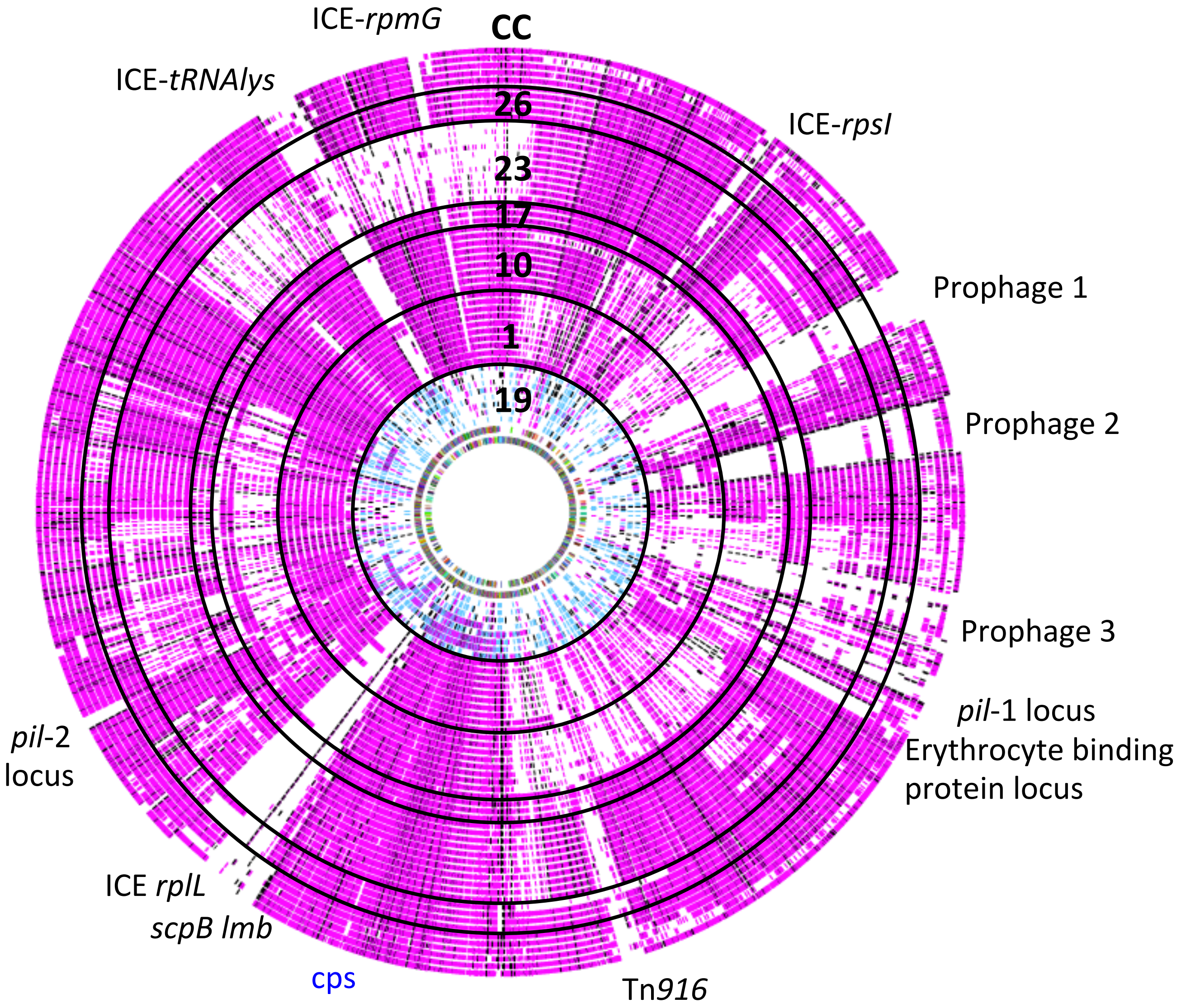

Supplementary Fig. 1: *Distribution of SNPs and recombination across 52 representative GBS isolates*. Circular view of SNPs across the whole genome. Isolates were selected to represent all major recombination events identified within each CC. The reference genome is the serotype III ST19 strain RBH11. Genomic islands from the reference genome are indicated in the outside circle as well as the capsular *cps* locus. Isolates were grouped by CCs delineated by black circles and their number indicated. SNPs are indicated by short lines on each circle. The variation in the SNP density reflects recombined regions. The figure was generated by using the SyntView software at http://genopole.pasteur.fr/SynTView/flash/ Streptococcus_agalactiae.

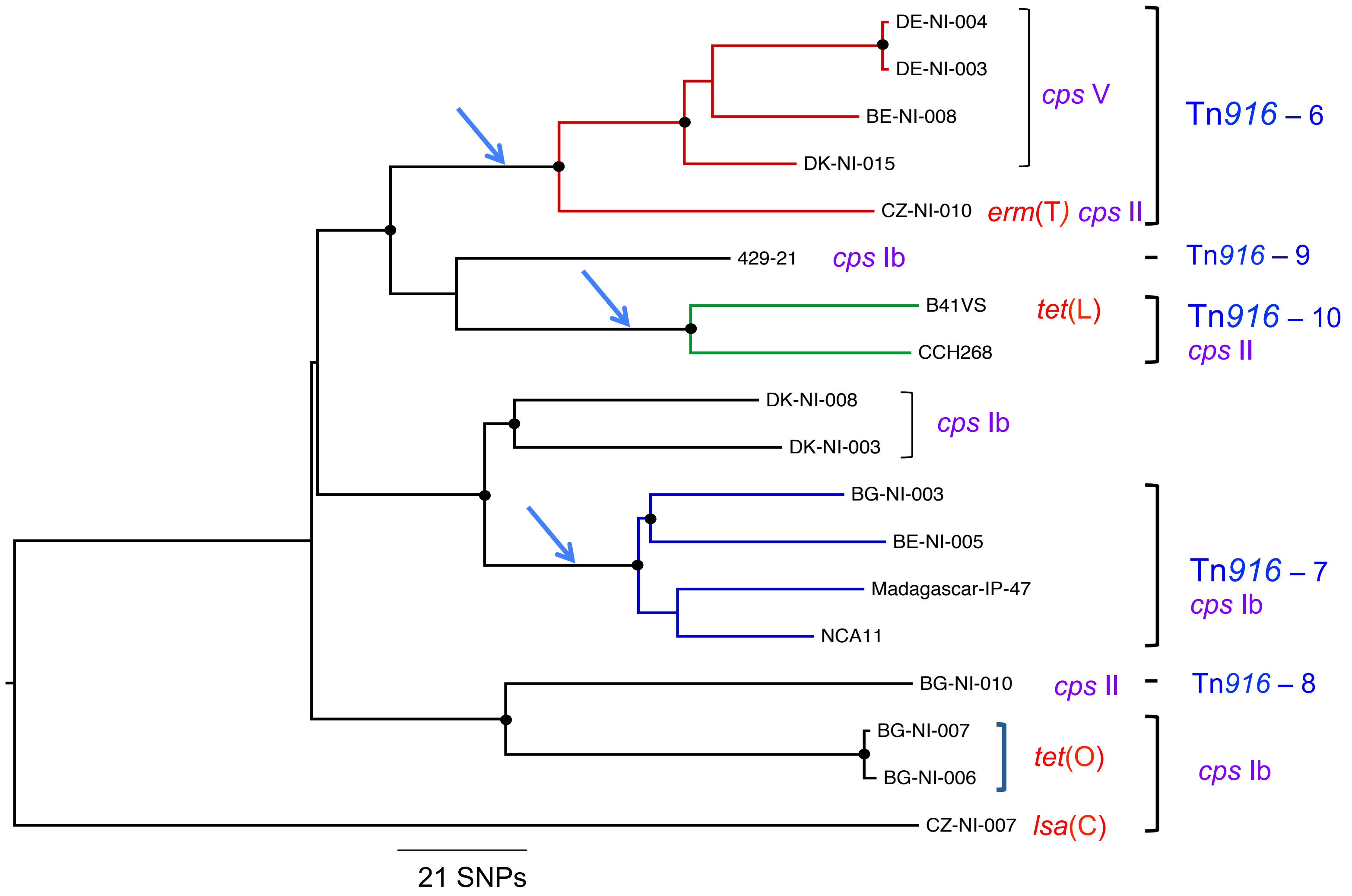

Supplementary Fig. 2: *Phylogeny of clonal complex CC10*. ML phylogeny from the alignment of pseudosequences of the 971 polymorphic positions in 1069 interrogated kbases. The previously defined clonal complexes 6, 8 and 10 were analysed together as they are closely related and named here CC10. The five independent Tn*916* or Tn*5801* insertions are indicated in blue and numbers from 6 to 10 refer to their description in Table 4. The three TcR lineages with more than one isolate are coloured in red, green and blue. Serotypes (*cps*) Ib, II and V are indicated in violet. Antibiotic resistance genes other than *tet*(M) are indicated in red. Nodes with >90% bootstrap support are indicated by black dots.

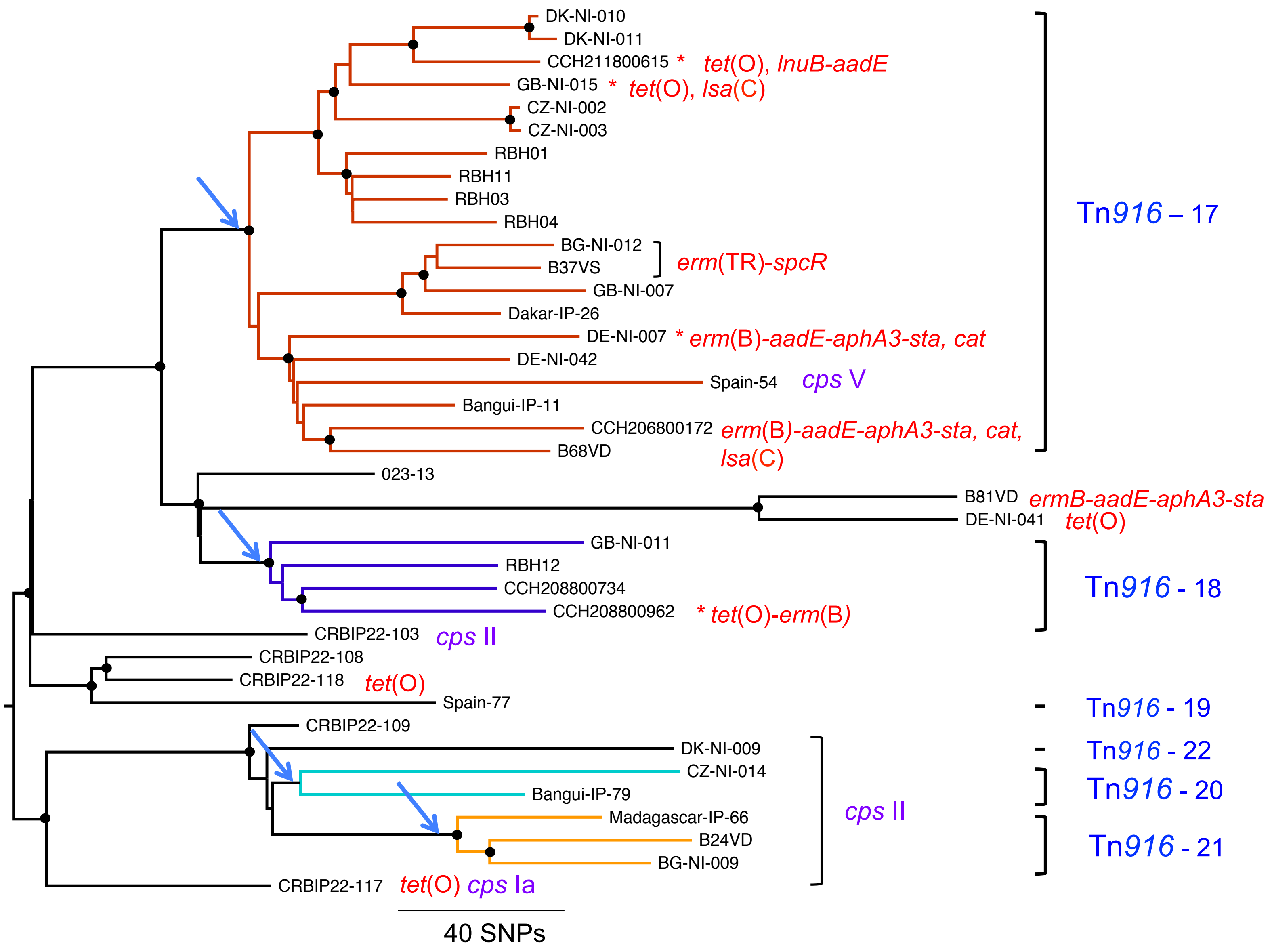

Supplementary Fig. 3: *Phylogeny of clonal complex CC19*. ML phylogeny from the alignment of pseudosequences of the 2016 polymorphic positions in 1427 interrogated kbases. The six independent Tn*916* or Tn*5801* insertions are indicated in blue and numbers from 17 to 22 refer to their description in Table 4. The four TcR lineages with more than one isolate are coloured in red, blue, turquoise and orange. Four isolates indicated by a star are predicted to have lost Tn*916*. The majority of the isolates are of serotype III. Other serotypes (*cps* Ia, II and V) are indicated in violet. Antibiotic resistance genes other than *tet*(M) are indicated in red. Nodes with >90% bootstrap support are indicated by black dots.

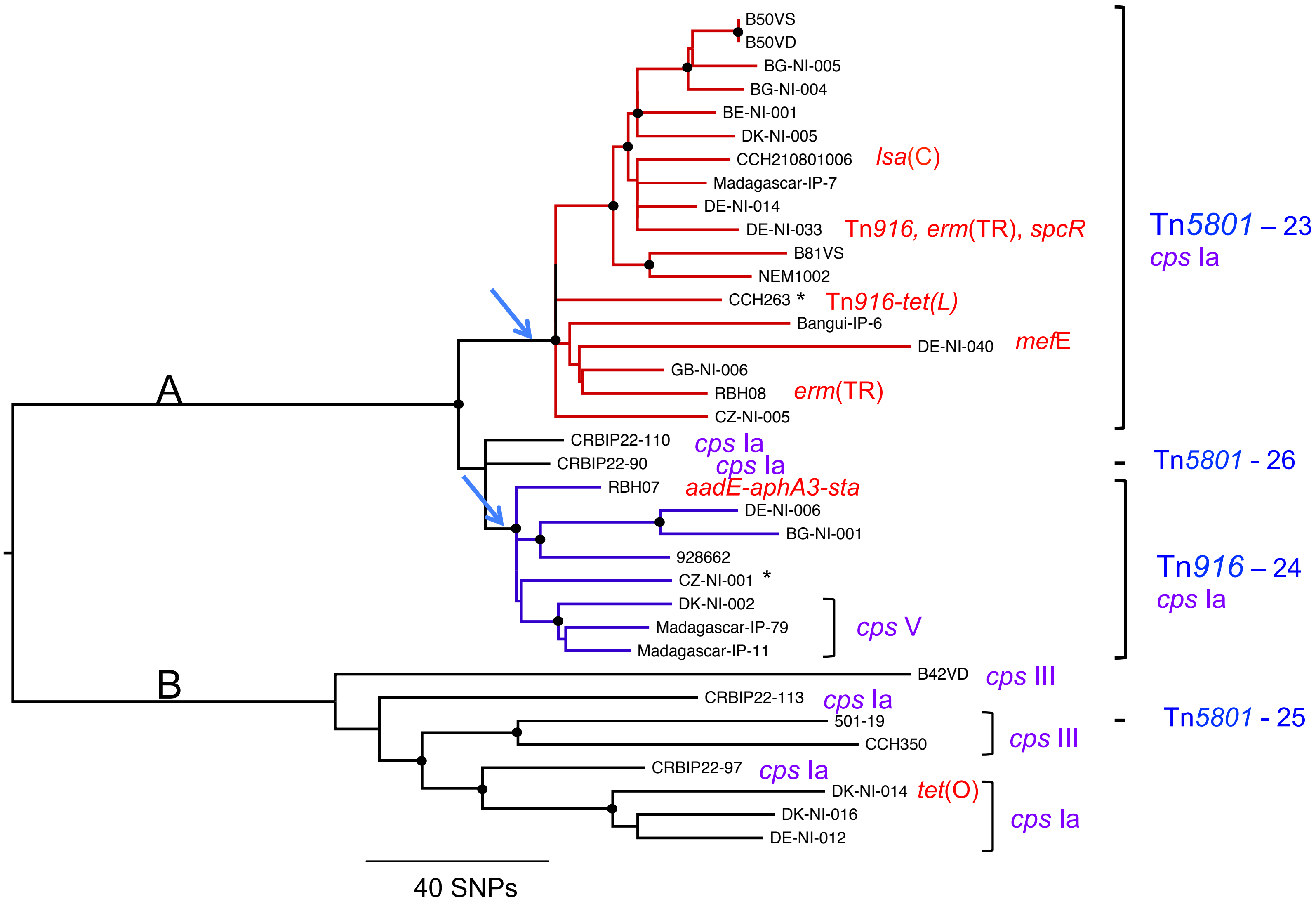

Supplementary Fig. 4: *Phylogeny of clonal complex CC23*. ML phylogeny from the alignment of pseudosequences of the 1329 polymorphic positions in 980 interrogated kbases. The four independent Tn*916* or Tn*5801* insertions are indicated in blue and numbers from 23 to 26 refer to their description in Table 4. The two TcR lineages with more than one isolate are coloured in red and blue. Serotypes (*cps*) Ia, III and V are indicated in violet The two major ST23 subdivisions A and B were previously identified following the sequencing of 15 genes. Cluster A comprises mostly human *cps* type Ia isolates. Cluster B were described to correspond mostly to bovine *cps* type III isolates, here exemplified by strain 501-19. However, most human isolates belonging to cluster B analysed here are of serotype Ia indicative of different events of capsular switches. Antibiotic resistance genes other than *tet*(M) are indicated in red. Nodes with >90% bootstrap support are indicated by black dots.

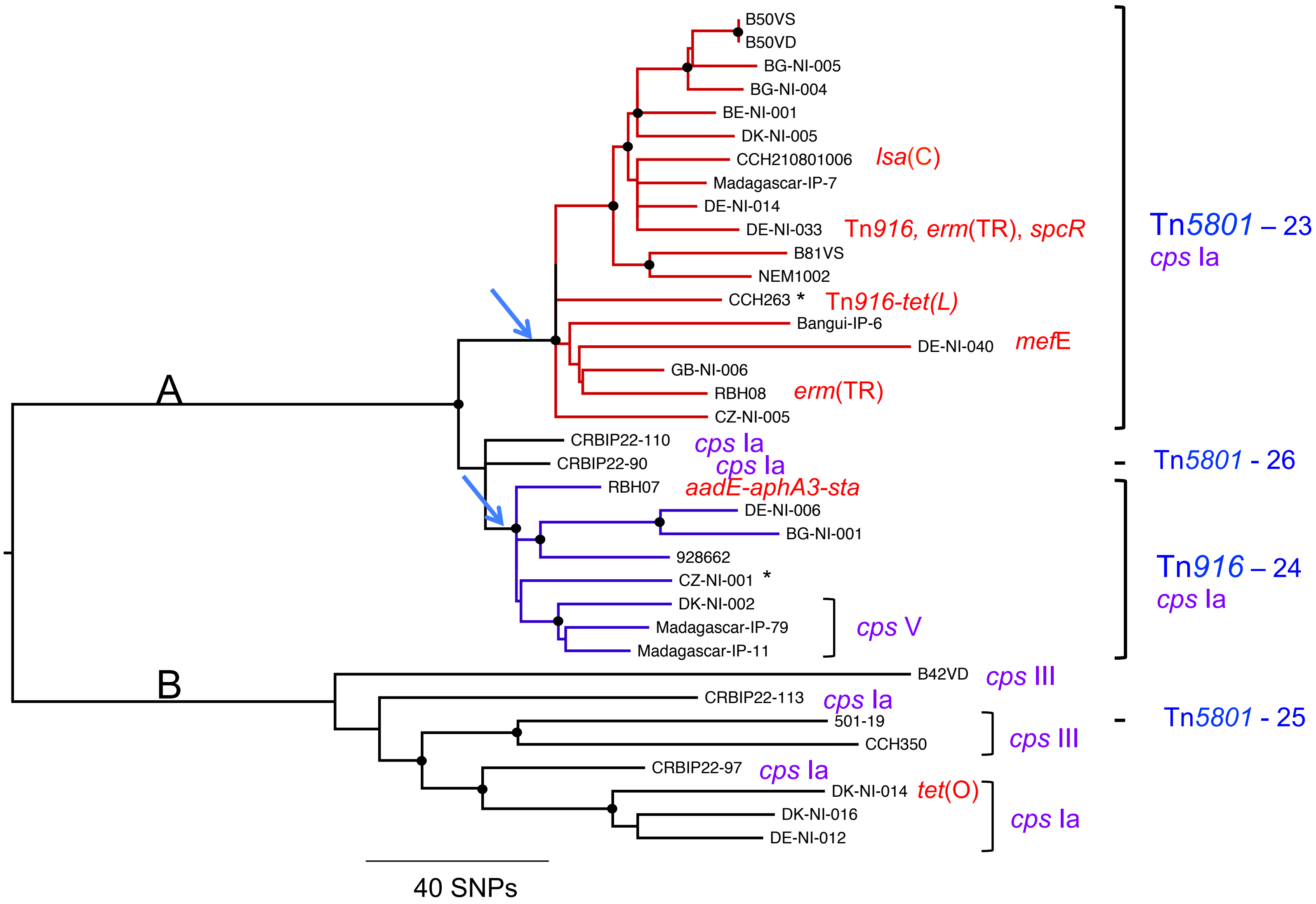

Supplementary Fig. 4: *Phylogeny of clonal complex CC23*. ML phylogeny from the alignment of pseudosequences of the 1329 polymorphic positions in 980 interrogated kbases. The four independent Tn*916* or Tn*5801* insertions are indicated in blue and numbers from 23 to 26 refer to their description in Table 4. The two TcR lineages with more than one isolate are coloured in red and blue. Serotypes (*cps*) Ia, III and V are indicated in violet The two major ST23 subdivisions A and B were previously identified following the sequencing of 15 genes. Cluster A comprises mostly human *cps* type Ia isolates. Cluster B were described to correspond mostly to bovine *cps* type III isolates, here exemplified by strain 501-19. However, most human isolates belonging to cluster B analysed here are of serotype Ia indicative of different events of capsular switches. Antibiotic resistance genes other than *tet*(M) are indicated in red. Nodes with >90% bootstrap support are indicated by black dots.

Supplementary Table 1

CC	Strain Name	Geographical origin	Year	Host	Clinical origin§	CPS type	ST	Antibiotic resistance gene(s)	TcR ICE Group	Read length	Sample accession number	Study accession number
CC1	DE-NI-022	Germany	2009	Human	NInv-EOD	V	ST387	*tet*(M)	G1-Tn*916*	PE 2 x 75	ERS039659	ERP000746
CC1	BG-NI-011	Bulgaria	2010	Human	NCarriage	V	ST1	*tet*(M)	G1-Tn*916*	PE 2 x 75	ERS039644	ERP000746
CC1	IT-NI-028	Italy	2009	Human	NInv-EOD	V	ST1	*tet*(M)	G1-Tn*916*	PE 2 x 75	ERS039675	ERP000746
CC1	GB-NI-009	Great Britain	2010	Human	NInv-EOD	V	ST1	*tet*(M)	G1-Tn*916*	PE 2 x 75	ERS039703	ERP000746
CC1	CZ-NI-006	Czech Republic	2008	Human	NCarriage	V	ST1	tet(M) *erm*(TR)	G1-Tn*916*	PE 2 x 75	ERS039687	ERP000746
CC1	CZ-NI-008	Czech Republic	2008	Human	NCarriage	V	ST1	*tet*(M)	G1-Tn*916*	PE 2 x 75	ERS039689	ERP000746
CC1	CZ-NI-009	Czech Republic	2008	Human	NCarriage	V	ST1	*tet*(M)	G1-Tn*916*	PE 2 x 75	ERS039690	ERP000746
CC1	B09PS	Australia	2008	Human	Carriage	V	ST1	*tet*(M)	G1-Tn*916*	PE 2 x 75	ERS046905	ERP000746
CC1	RBH05	Australia	2008	Human	Carriage	V	ST1	*tet*(M)	G1-Tn*916*	PE 2 x 75	ERS046920	ERP000746
CC1	RBH09	Australia	2008	Human	Carriage	V	ST1	*tet*(M)	G1-Tn*916*	PE 2 x 75	ERS046924	ERP000746
CC1	CCH206800162	France	2006	Human	AInvD	V	ST1	*tet*(M)	G1-Tn*916*	110	ERS337526	PRJEB4456
CC1	CCH208800538	France	2008	Human	InvD	V	ST1	*tet(M) erm(TR)*	G1-Tn*916*	110	ERS337529	PRJEB4456
CC1	p5465	Germany	1997	Dog	Carriage	V	ST1	*tet*(M)	G1-Tn*916*	51	ERS337455	PRJEB4456
CC1	IT-NI-0031	Italy	2010	Human	NInv-EOD	V	ST1	*erm*(TR)	G1-Tn*916*	PE 2 x 75	ERS039676	ERP000746
CC1	DK-NI-012	Denmark	2010	Human	NInv-EOD	V	ST1	*tet*(M) *erm*(B)	G1-Tn*916*	PE 2 x 75	ERS039627	ERP000746
CC1	DK-NI-013	Denmark	2010	Human	NCarriage	V	ST1	*tet*(M) *erm*(B)	G1-Tn*916*	PE 2 x 75	ERS039628	ERP000746
CC1	DK-NI-022	Denmark	2011	Human	NInv-EOD	V	ST1	*tet*(M) *erm*(B)	G1-Tn*916*	PE 2 x 75	ERS039634	ERP000746
CC1	DE-NI-001	Germany	2007	Human	NInv-EOD	V	ST1	*tet*(M) *erm*(B)	G1-Tn*916*	PE 2 x 75	ERS039648	ERP000746
CC1	CZ-NI-004	Czech Republic	2008	Human	NCarriage	V	ST1	*tet*(M) *erm*(B)	G1-Tn*916*	PE 2 x 75	ERS039685	ERP000746
CC1	CZ-NI-015	Czech Republic	2009	Human	NInv-EOD	V	ST1	*tet*(M) *erm*(B)	G1-Tn*916*	PE 2 x 75	ERS039694	ERP000746
CC1	GB-NI-010	Great Britain	2010	Human	NInv-EOD	V	ST1	*tet*(M) *erm*(B)	G1-Tn*916*	PE 2 x 75	ERS039704	ERP000746
CC1	CZ-NI-013	Czech Republic	2009	Human	NCarriage	V	ST1	*tet*(M) *erm*(B)	G1-Tn*916*	PE 2 x 75	ERS039692	ERP000746
CC1	CCH239	France	1998	Human	urine	V	ST1	*tet*(M) *erm*(B)	G1-Tn*916*	51	ERS337453	PRJEB4456
CC1	Bangui-IP-50	C.A.F$	2006	Human	NA	V	ST1	*tet*(M) *erm*(B)	G1-Tn*916*	51	ERS337451	PRJEB4456

Supplementary Table 1

CC1	CCH208800262	France	2008	Human	AInvD	V	ST1	*tet*(M) *erm*(B)	G1-Tn*916*	110	ERS337528	PRJEB4456
CC1	Bangui-IP-59	C. A. F. $	2007	Human	NA	V	ST1	*tet*(M) *erm*(B)	G1-Tn*916*	51	ERS337524	PRJEB4456
CC1	Spain-IP-68	Spain	2005	Human	NA	V	ST1		G1-Tn*916*	51	ERS337437	PRJEB4456
CC1	DK-NI-019	Denmark	2010	Human	NCarriage	IV	ST196	*tet*(M)	G2-Tn*916*	PE 2 x 75	ERS039632	ERP000746
CC1	CZ-NI-016	Czech Republic	2010	Human	NInv-EOD	IV	ST459-like#	*tet*(M) *erm* (TR) *spcR*	G2-Tn*916*	PE 2 x 75	ERS039695	ERP000746
CC1	CCH207800464	France	2007	Human	NA	IV	ST196	*tet*(M)	G2-Tn*916*	101	ERS337456	PRJEB4456
CC1	CCH209800741	France	2009	Human	AInvD	IV	ST196	*tet*(M)	G2-Tn*916*	101	ERS337457	PRJEB4456
CC1	CCH210800418	France	2010	Human	AInvD	IV	ST459	*tet*(M) *erm* (TR) *spcR*	G2-Tn*916*	101	ERS337460	PRJEB4456
CC1	CCH208800621	France	2008	Human	Carriage	IV	ST459	*tet*(M) *erm* (TR) *spcR*	G2-Tn*916*	101	ERS337459	PRJEB4456
CC1	RBH02	Australia	2008	Human	Carriage	IV	ST2	*tet*(M)	G3-Tn*5801*	PE 2 x 75	ERS046917	ERP000746
CC1	Bangui-IP-8	C. A. F. $	2007	Human	Carriage	IV	ST2	*tet*(M)	G4-Tn*5801*	101	ERS337458	PRJEB4456
CC1	DE-NI-010	Germany	2008	Human	NInv-EOD	II	ST2	*tet*(M)	G5-Tn*916*	PE 2 x 75	ERS039653	ERP000746
CC1	CCH330	France	1998	Human	urine	VI	ST1			51	ERS337454	PRJEB4456
CC1	CCH208800620	France	2008	Human	Carriage	V	ST1	*lsa*(C)		110	ERS337452	PRJEB4456
CC1	Spain-IP-33	Spain	2005	Bovine	Bovine milk	II	ST2-like&	*tet*(O) *aadE* *erm*(B)		51	ERS337438	PRJEB4456
CC10	DK-NI-015	Denmark	2008	Human	NCarriage	V	ST10-like&	*tet*(M)	G6-Tn*5801*	PE 2 x 75	ERS039630	ERP000746
CC10	BE-NI-008	Belgium	2010	Human	Carriage	V	ST10	*tet*(M)	G6-Tn*5801*	PE 2 x 75	ERS039616	ERP000746
CC10	DE-NI-003	Germany	2008	Human	NInv-EOD	V	ST10-like#-&	*tet*(M)	G6-Tn*5801*	PE 2 x 75	ERS039649	ERP000746
CC10	DE-NI-004	Germany	2008	Human	NInv-EOD	V	ST10	*tet*(M)	G6-Tn*5801*	PE 2 x 75	ERS039650	ERP000746
CC10	CZ-NI-010	Czech Republic	2009	Human	NCarriage	II	ST10-like#	*tet*(M) *erm*(T)	G6-Tn*5801*	PE 2 x 75	ERS039691	ERP000746
CC10	BE-NI-005	Belgium	2010	Human	Carriage	Ib	ST8	*tet*(M)	G7-Tn*916*	PE 2 x 75	ERS039614	ERP000746
CC10	BG-NI-003	Bulgaria	2009	Human	NCarriage	Ib	ST8	*tet*(M)	G7-Tn*916*	PE 2 x 75	ERS039637	ERP000746
CC10	Madagascar-IP-47	Madagascar	2007	Human	Carriage	Ib	ST8-like&	*tet*(M)	G7-Tn*916*	110	ERS337547	PRJEB4456
CC10	NCA11	New Caledonia	2003	Human	Carriage	Ib	ST8-like&	*tet*(M)	G7-Tn*916*	110	ERS337535	PRJEB4456
CC10	BG-NI-010	Bulgaria	2009	Human	NCarriage	II	ST12-like#-&	*tet*(M)	G8-Tn*916*	PE 2 x 75	ERS039643	ERP000746

Supplementary Table 1

CC10	429_21	France	1983	Bovine	Bovine Mastitis	Ib	ST10	*tet*(M)	G9-Tn*916*	51	ERS337549	PRJEB4456
CC10	B41VS	Australia	2008	Human	Carriage	II	ST10-like[&]	*tet*(M) *tet*(L)	G10-Tn*916*	PE 2 x 75	ERS046928	ERP000746
CC10	CCH268	France	1998	Human	Sperm	II	ST10	*tet*(M)	G10-Tn*916*	51	ERS337542	PRJEB4456
CC10	DK-NI-008	Denmark	2009	Human	NCarriage	Ib	ST9-like[#-&]			PE 2 x 75	ERS039623	ERP000746
CC10	DK-NI-003	Denmark	2009	Human	NCarriage	Ib	ST523-like[#-&]			PE 2 x 75	ERS039619	ERP000746
CC10	BG-NI-006	Bulgaria	2009	Human	NCarriage	Ib	ST12-like[#]	*tet*(O)		PE 2 x 75	ERS039640	ERP000746
CC10	BG-NI-007	Bulgaria	2009	Human	NCarriage	Ib	ST12-like[&]	*tet*(O)		PE 2 x 75	ERS039641	ERP000746
CC10	CZ-NI-007	Czech Republic	2008	Human	NCarriage	Ib	ST255-like[#]	*lsa*(C)		PE 2 x 75	ERS039688	ERP000746
CC17	DK-NI-007	Denmark	2009	Human	NInv-EOD	III	ST17-like[#]	*tet*(M)	G11-Tn*5801*	PE 2 x 75	ERS039622	ERP000746
CC17	DK-NI-021	Denmark	2009	Human	NCarriage	III	ST17-like[#]	*tet*(M)	G11-Tn*5801*	PE 2 x 75	ERS039633	ERP000746
CC17	BG-NI-002	Bulgaria	2009	Human	NCarriage	III	ST17-like[#]	*tet*(M)	G11-Tn*5801*	PE 2 x 75	ERS039636	ERP000746
CC17	DK-NI-001	Denmark	2009	Human	NCarriage	III	ST17-like[#]	*tet*(M)	G11-Tn*5801*	PE 2 x 75	ERS039617	ERP000746
CC17	DK-NI-004	Denmark	2009	Human	NInv-EOD	III	ST17-like[#]	*tet*(M)	G11-Tn*5801*	PE 2 x 75	ERS039620	ERP000746
CC17	DE-NI-032	Germany	2009	Human	NInv-EOD	III	ST17-like[#]	*tet*(M)	G11-Tn*5801*	PE 2 x 75	ERS039660	ERP000746
CC17	IT-NI-009	Italy	2008	Human	NInv-EOD	III	ST17-like[#-&]	*tet*(M)	G11-Tn*5801*	PE 2 x 75	ERS039670	ERP000746
CC17	IT-NI-018	Italy	2009	Human	NInv-EOD	III	ST17-like[#]	*tet*(M)	G11-Tn*5801*	PE 2 x 75	ERS039672	ERP000746
CC17	IT-NI-019	Italy	2009	Human	NInv-EOD	III	ST17-like[#]	*tet*(M)	G11-Tn*5801*	PE 2 x 75	ERS039673	ERP000746
CC17	IT-NI-020	Italy	2009	Human	NInv-EOD	III	ST17-like[#]	*tet*(M)	G11-Tn*5801*	PE 2 x 75	ERS039674	ERP000746
CC17	IT-NI-032	Italy	2010	Human	NInv-EOD	III	ST17-like[#]	*tet*(M)	G11-Tn*5801*	PE 2 x 75	ERS039677	ERP000746
CC17	IT-NI-033	Italy	2010	Human	NInv-EOD	III	ST17-like[#]	*tet*(M)	G11-Tn*5801*	PE 2 x 75	ERS039678	ERP000746
CC17	IT-NI-034	Italy	2010	Human	NInv-EOD	III	ST17-like[#]	*tet*(M)	G11-Tn*5801*	PE 2 x 75	ERS039679	ERP000746
CC17	DE-NI-036	Germany	2010	Human	NInv-EOD	III	ST17-like[#]	*tet*(M)	G11-Tn*5801*	PE 2 x 75	ERS039662	ERP000746
CC17	DE-NI-037	Germany	2010	Human	NInv-EOD	III	ST17-like[#]	*tet*(M)	G11-Tn*5801*	PE 2 x 75	ERS039663	ERP000746
CC17	IT-NI-007	Italy	2008	Human	NInv-EOD	III	ST17-like[#]	*tet*(M)	G11-Tn*5801*	PE 2 x 75	ERS039668	ERP000746
CC17	IT-NI-008	Italy	2008	Human	NInv-EOD	III	ST17-like[#]	*tet*(M)	G11-Tn*5801*	PE 2 x 75	ERS039669	ERP000746

Supplementary Table 1

CC17	GB-NI-003	Great Britain	2009	Human	NInv-EOD	III	ST17-like[#]	*tet*(M)	G11-Tn*5801*	PE 2 x 75	ERS039698	ERP000746
CC17	GB-NI-004	Great Britain	2010	Human	NInv-EOD	III	ST17-like[#]	*tet*(M)	G11-Tn*5801*	PE 2 x 75	ERS039699	ERP000746
CC17	GB-NI-005	Great Britain	2009	Human	NInv-EOD	III	ST17-like[#-&]	*tet*(M)	G11-Tn*5801*	PE 2 x 75	ERS039700	ERP000746
CC17	B15VD	Australia	2008	Human	Carriage	III	ST17		G11-Tn*5801*	PE 2 x 75	ERS046906	ERP000746
CC17	CCH208800879	France	2008	Human	NInv-LOD	III	ST17	*tet*(M)	G11-Tn*5801*	101	ERS337471	PRJEB4456
CC17	CCH210800688	France	2010	Human	NInv-LOD	III	ST17	*tet*(M)	G11-Tn*5801*	110	ERS337491	PRJEB4456
CC17	CCH210125551	France	2010	Human	Carriage	III	ST17	*tet*(M)	G11-Tn*5801*	51	ERS337478	PRJEB4456
CC17	CCH208800031	France	2008	Human	NInv-LOD	III	ST17	*tet*(M)	G11-Tn*5801*	51	ERS337467	PRJEB4456
CC17	CCH207800974	France	2007	Human	LOD	III	ST17	*tet*(M) *tet*(O) *erm*(B)	G11-Tn*5801*	101	ERS337466	PRJEB4456
CC17	CCH206800391	France	2006	Human	NInv-LOD	III	ST17	*tet*(M)	G11-Tn*5801*	110	ERS337462	PRJEB4456
CC17	CCH210150530	France	2010	Human	Carriage	III	ST17	*tet*(M)	G11-Tn*5801*	51	ERS337481	PRJEB4456
CC17	CCH210800096	France	2010	Human	NInv-LOD	III	ST17	*tet*(M)	G11-Tn*5801*	51	ERS337488	PRJEB4456
CC17	CCH210169151	France	2010	Human	Carriage	III	ST17	*tet*(M)	G11-Tn*5801*	51	ERS337485	PRJEB4456
CC17	CCH208800438	France	2008	Human	NInv-LOD	III	ST17-like[#]	*tet*(M)	G11-Tn*5801*	51	ERS337469	PRJEB4456
CC17	CCH210160764	France	2010	Human	NInv-LOD	III	ST17	*tet*(M) *erm*(B)	G11-Tn*5801*	51	ERS337484	PRJEB4456
CC17	CCH209800522	France	2009	Human	NInv-LOD	III	ST17	*tet*(M)	G11-Tn*5801*	51	ERS337475	PRJEB4456
CC17	CCH208800147	France	2008	Human	NInv-LOD	III	ST17		G11-Tn*5801*	51	ERS337468	PRJEB4456
CC17	NEM318	France	1995	Human	NInv-LOD	III	ST17	*tet*(M)	G11-Tn*5801*	36	ERS337504	PRJEB4456
CC17	CCH210152823	France	2010	Human	Carriage	III	ST17	*tet*(M)	G11-Tn*5801*	51	ERS337482	PRJEB4456
CC17	CCH206800353	France	2006	Human	NInv-LOD	III	ST17	*tet*(M)	G11-Tn*5801*	51	ERS337461	PRJEB4456
CC17	CCH210800593	France	2008	Human	NInv-LOD	III	ST17	*tet*(M)	G11-Tn*5801*	51	ERS337490	PRJEB4456
CC17	CCH209800918	France	2009	Human	NInv-LOD	III	ST17	*tet*(M)	G11-Tn*5801*	101	ERS337493	PRJEB4456
CC17	Dakar IP-107	C. A. F.[$]	2007	Human	Carriage	III	ST17	*tet*(M)	G11-Tn*5801*	110	ERS337532	PRJEB4456
CC17	Bangui-IP-61	C. A. F.[$]	2007	Human	Carriage	III	ST17	*tet*(M)	G11-Tn*5801*	51	ERS337525	PRJEB4456
CC17	COH1	USA		Human	InvD	III	ST17	*tet*(M)	G11-Tn*5801*	Sanger	HG939456	
CC17	BE-NI-007	Belgium	2010	Human	Carriage	III	ST315-like[#]	*tet*(M)	G12-Tn*916*	PE 2 x 75	ERS039615	ERP000746
CC17	DE-NI-0013	Germany	2009	Human	NInv-EOD	III	ST17-like[#]	*tet*(M)	G12-Tn*916*	PE 2 x 75	ERS039655	ERP000746

Supplementary Table 1

CC17	DE-NI-0017	Germany	2009	Human	NInv-EOD	III	ST17-like[#]	*tet*(M)	G12-Tn*916*	PE 2 x 75	ERS039657	ERP000746
CC17	DE-NI-0019	Germany	2009	Human	NInv-EOD	III	ST17-like[#]	*tet*(M)	G12-Tn*916*	PE 2 x 75	ERS039658	ERP000746
CC17	B96P	Australia	2008	Human	Carriage	III	ST17	*tet*(M)	G12-Tn*916*	PE 2 x 75	ERS046927	ERP000746
CC17	B96V	Australia	2008	Human	Carriage	III	ST17	*tet*(M)	G12-Tn*916*	PE 2 x 75	ERS046932	ERP000746
CC17	CCH210169294	France	2010	Human	Carriage	III	ST17	*tet*(M)	G12-Tn*916*	51	ERS337486	PRJEB4456
CC17	CCH209800160	France	2009	Human	NInv-LOD	III	ST17	*tet*(M)	G12-Tn*916*	51	ERS337473	PRJEB4456
CC17	CCH210126535	France	2010	Human	Carriage	III	ST17	*tet*(M)	G12-Tn*916*	51	ERS337479	PRJEB4456
CC17	CCH208800481	France	2008	Human	NInv-LOD	III	ST17	*tet*(M)	G12-Tn*916*	51	ERS337470	PRJEB4456
CC17	CCH210155112	France	2010	Human	Carriage	III	ST17	*tet*(M)	G12-Tn*916*	51	ERS337483	PRJEB4456
CC17	CCH209800039	France	2009	Human	NInv-LOD	III	ST17	*tet*(M)	G12-Tn*916*	51	ERS337472	PRJEB4456
CC17	CCH207800343	France	2007	Human	NInv-LOD	III	ST17	*tet*(M)	G12-Tn*916*	51	ERS337465	PRJEB4456
CC17	CRBIP22.120	France	1961	Human	InvD	III	ST17-like[#]	*tet*(M)	G12-Tn*916*	PE 2 x 75	ERS337494	ERP000746
CC17	BM110	USA	<1989	Human	Ninv	III	ST17	*tet*(M)	G12-Tn*916*	50	ERS337464	PRJEB4456
CC17	Madagascar-IP-33	Madagascar	2006	Human	NA	III	ST17-like[#]	*tet*(M)	G12-Tn*916*	51	ERS337500	PRJEB4456
CC17	CRBIP22.94	France	1955	Human	NA	III	ST17	*tet*(M)	G12-Tn*916*	51	ERS337495	PRJEB4456
CC17	CCH210107206	France	2010	Human	Carriage	III	ST17	*tet*(M)	G12-Tn*916*	51	ERS337477	PRJEB4456
CC17	CCH209801071	France	2009	Human	NInv-LOD	III	ST17-like[#]	*tet*(M)	G12-Tn*916*	51	ERS337476	PRJEB4456
CC17	CRBIP22.98	France	1955	Human	NA	III	ST17	*tet*(M)	G12-Tn*916*	51	ERS337496	PRJEB4456
CC17	WC3/UK21	UK	2001	Human	NInv-EOD	III	ST17	*tet*(M)	G12-Tn*916*	36	ERS337507	PRJEB4456
CC17	Dakar-IP-8	Senegal	2007	Human	Carriage	III	ST17	*tet*(M)	G12-Tn*916*	51	ERS337534	PRJEB4456
CC17	CZ183	Czech republic		Animal	Bovine	III	ST355-like[#]	*tet*(M)	G12-Tn*916*	36	ERS337497	PRJEB4456
CC17	NEM1857	France	2001	Human	NInv-LOD	III	ST252-like[&]	*tet*(M)	G13-Tn*916*	51	ERS337503	PRJEB4456
CC17	CCH210172343	France	2010	Human	Carriage	III	ST17	*tet*(M)	G13-Tn*916*	51	ERS337487	PRJEB4456
CC17	CCH210800140	France	2010	Human	NInv-LOD	III	ST17	*tet*(M)	G13-Tn*916*	51	ERS337489	PRJEB4456
CC17	Dakar-IP-98	Senegal	2007	Human	Carriage	III	ST17	*tet*(M)	G13-Tn*916*	51	ERS337498	PRJEB4456
CC17	Bangui-IP-29	C. A. F.[$]	2007	Human	Carriage	III	ST17	*tet*(M)	G13-Tn*916*	110	ERS337523	PRJEB4456
CC17	Madagascar-IP-84	Madagascar	2007	Human	Carriage	III	ST17	*tet*(M)	G13-Tn*916*	51	ERS337502	PRJEB4456

Supplementary Table 1

CC17	CCH210145919	France	2010	Human	Carriage	III	ST17	*tet*(M)	G14-Tn*916*	51	ERS337480	PRJEB4456
CC17	Madagascar-IP-71	Madagascar	2007	Human	Carriage	III	ST17-like[#]	*tet*(M)	G14-Tn*916*	51	ERS337501	PRJEB4456
CC17	Bangui-IP-112	C. A. F.[$]	2007	Human	Carriage	III	ST17	*tet*(M)	G15-Tn*916*	51	ERS337522	PRJEB4456
CC17	Madagascar-IP-20	Madagascar	2006	Human	NA	IV	ST291	*tet*(M)	G16-Tn*5801*	101	ERS337499	PRJEB4456
CC17	CCH211800247	France	2011	Human	AInv arthritis	IV	ST291	*tet*(M)	G16-Tn*5801*	101	ERS337541	PRJEB4456
CC17	CCH209800361	France	2009	Human	AInvD	IV	ST291	*tet*(M)	G16-Tn*5801*	101	ERS337474	PRJEB4456
CC17	Spain-82	Spain	2005	Human	NA	IV	ST291	*tet*(M)	G16-Tn*5801*	101	ERS337506	PRJEB4456
CC17	CCH211800398	France	2011	Human	Carriage	IV	ST291	*tet*(M)	G16-Tn*5801*	101	ERS337492	PRJEB4456
CC19	DK-NI-010	Denmark	2009	Human	NInv-EOD	III	ST19	*tet*(M)	G17-Tn*916*	PE 2 x 75	ERS039625	ERP000746
CC19	DK-NI-011	Denmark	2009	Human	NCarriage	III	ST19-like[#-&]	*tet*(M)	G17-Tn*916*	PE 2 x 75	ERS039626	ERP000746
CC19	DE-NI-007	Germany	2008	Human	NInv-EOD	III	ST19	*erm*(B) *aadE aphA3 sta cat*	G17-Tn*916*	PE 2 x 75	ERS039652	ERP000746
CC19	BG-NI-012	Bulgaria	2010	Human	NInv-EOD	III	ST19	*tet*(M) *erm*(TR) *spcR*	G17-Tn*916*	PE 2 x 75	ERS039645	ERP000746
CC19	CZ-NI-002	Czech Republic	2008	Human	NCarriage	III	ST19	*tet*(M)	G17-Tn*916*	PE 2 x 75	ERS039683	ERP000746
CC19	CZ-NI-003	Czech Republic	2008	Human	NCarriage	III	ST19	*tet*(M)	G17-Tn*916*	PE 2 x 75	ERS039684	ERP000746
CC19	DE-NI-042	Germany	2010	Human	NInv-EOD	III	ST19-like[#-&]	*tet*(M)	G17-Tn*916*	PE 2 x 75	ERS039666	ERP000746
CC19	GB-NI-007	Great Britain	2010	Human	NInv-EOD	III	ST19	*tet*(M)	G17-Tn*916*	PE 2 x 75	ERS039702	ERP000746
CC19	GB-NI-015	Great Britain	2010	Human	NInv-EOD	III	ST19-like[#-&]	*tet*(O) *lsa*(C)	G17-Tn*916*	PE 2 x 75	ERS039707	ERP000746
CC19	RBH01	Australia	2008	Human	Carriage	III	ST19	*tet*(M)	G17-Tn*916*	PE 2 x 75	ERS046916	ERP000746
CC19	RBH03	Australia	2008	Human	Carriage	III	ST19	*tet*(M)	G17-Tn*916*	PE 2 x 75	ERS046918	ERP000746
CC19	RBH04	Australia	2008	Human	Carriage	III	ST19	*tet*(M)	G17-Tn*916*	PE 2 x 75	ERS046919	ERP000746
CC19	RBH11	Australia	2008	Human	Carriage	III	ST19	*tet*(M)	G17-Tn*916*	PE 2 x 75	ERS046925	ERP000746
CC19	B37VS	Australia	2008	Human	Carriage	III	ST335	*tet*(M) *erm*(TR) *spcR*	G17-Tn*916*	PE 2 x 75	ERS046908	ERP000746
CC19	B68VD	Australia	2008	Human	Carriage	III	ST19-like[&]	*tet*(M)	G17-Tn*916*	PE 2 x 75	ERS046911	ERP000746
CC19	CCH211800615	France	2011	Human	Carriage	III	ST19	*tet*(O) *lnuB aadE*	G17-Tn*916*	110	ERS337441	PRJEB4456

Supplementary Table 1

CC19	Bangui-IP-11	C. A. F.$	2006	Human	NA	III	ST182	*tet*(M)	G17-Tn*916*	110	ERS337439	PRJEB4456
CC19	Spain-54	Spain	2005	Animal	Milk	V	ST19	*tet*(M)	G17-Tn*916*	51	ERS337449	PRJEB4456
CC19	Dakar-IP-26	Senegal	2007	Human	Carriage	III	ST19-like&	*tet*(M)	G17-Tn*916*	51	ERS337533	PRJEB4456
CC19	CCH206800172	France	2006	Human	InvD	III	ST19	*tet*(M) *lsa*(C) *erm*(B) *aadE aphA3 sta cat*	G17-Tn*916*	110	ERS337527	PRJEB4456
CC19	GB-NI-011	Great Britain	2010	Human	NCarriage	III	ST19	*tet*(M)	G18-Tn*916*	PE 2 x 75	ERS039705	ERP000746
CC19	RBH12	Australia	2008	Human	Carriage	III	ST19	*tet*(M)	G18-Tn*916*	PE 2 x 75	ERS046926	ERP000746
CC19	CCH208800962	France	2008	Human	AInvD	III	ST19	*tet*(O) *erm*(B)	G18-Tn*916*	110	ERS337531	PRJEB4456
CC19	CCH208800734	France	2008	Human	AInvD	III	ST19	*tet*(M)	G18-Tn*916*	110	ERS337530	PRJEB4456
CC19	Spain-77	Spain	2005	Human	Carriage	III	ST19	*tet*(M)	G19-Tn916	51	ERS337450	PRJEB4456
CC19	CZ-NI-014	Czech Republic	2009	Human	NInv-EOD	II	ST479-like#	*tet*(M)	G20-Tn*916*	PE 2 x 75	ERS039693	ERP000746
CC19	Bangui-IP-79	C. A. F.$	2006	Human	NA	II	ST28	*tet*(M)	G20-Tn*916*	110	ERS337444	PRJEB4456
CC19	BG-NI-009	Bulgaria	2008	Human	NCarriage	II	ST28-like#-&	*tet*(M)	G21-Tn*916*	PE 2 x 75	ERS039642	ERP000746
CC19	B24VD	Australia	2008	Human	Carriage	II	ST28	*tet*(M)	G21-Tn*916*	PE 2 x 75	ERS046907	ERP000746
CC19	Madagascar-IP-66	Madagascar	2006	Human	NA	II	ST28	*tet*(M)	G21-Tn*916*	51	ERS337448	PRJEB4456
CC19	DK-NI-009	Denmark	2009	Human	NCarriage	II	ST28	*tet*(M)	G22-Tn916	PE 2 x 75	ERS039624	ERP000746
CC19	CRBIP22.109	France	1959	Human	InvD	II	ST28			110	ERS337445	PRJEB4456
CC19	B81VD	Australia	2008	Human	Carriage	III	ST106	*erm(B) aadE aphA3 sta*		PE 2 x 75	ERS046930	ERP000746
CC19	023_13	France	1969	Goat	NA	III	ST19			51	ERS337440	PRJEB4456
CC19	CRBIP22.108	France	1958	Human	Carriage	III	ST19			110	ERS337443	PRJEB4456
CC19	CRBIP22.103	USA	1956	Human	NA	II	ST19			51	ERS337442	PRJEB4456
CC19	DE-NI-041	Germany	2008	Human	NInv-EOD	III	ST106	*tet*(O)		PE 2 x 75	ERS039665	ERP000746
CC19	CRBIP22.117	France	1960	Human	NA	Ia	ST19	*tet*(O)		110	ERS337446	PRJEB4456
CC19	CRBIP22.118	Denmark	1960	Human	Urine	III	ST19	*tet*(O)		110	ERS337447	PRJEB4456
CC23	BE-NI-001	Belgium	2009	Human	Carriage	Ia	ST23-like#	*tet*(M)	G23-Tn*5801*	PE 2 x 75	ERS039613	ERP000746
CC23	DK-NI-005	Denmark	2009	Human	NInv-EOD	Ia	ST23-like#	*tet*(M)	G23-Tn*5801*	PE 2 x 75	ERS039621	ERP000746

Supplementary Table 1

CC23	BG-NI-004	Bulgaria	2010	Human	NCarriage	Ia	ST23-like#	*tet*(M)	G23-Tn*5801*	PE 2 x 75	ERS039638	ERP000746
CC23	DE-NI-014	Germany	2009	Human	NInv-EOD	Ia	ST23-like#	*tet*(M)	G23-Tn*5801*	PE 2 x 75	ERS039656	ERP000746
CC23	BG-NI-005	Bulgaria	2009	Human	NInv-EOD	Ia	ST23-like#	*tet*(M)	G23-Tn*5801*	PE 2 x 75	ERS039639	ERP000746
CC23	DE-NI-033	Germany	2010	Human	NInv-EOD	Ia	ST23-like#	*tet*(M) *tet*(M) *erm*(TR) *spcR*	G23-Tn*5801*	PE 2 x 75	ERS039661	ERP000746
CC23	DE-NI-040	Germany	2010	Human	NInv-EOD	Ia	ST23-like#	*tet*(M) *mefE*	G23-Tn*5801*	PE 2 x 75	ERS039664	ERP000746
CC23	GB-NI-006	Great Britain	2010	Human	NInv-EOD	Ia	ST23	*tet*(M)	G23-Tn*5801*	PE 2 x 75	ERS039701	ERP000746
CC23	CZ-NI-005	Czech Republic	2008	Human	NCarriage	Ia	ST23-like#	*tet*(M)	G23-Tn*5801*	PE 2 x 75	ERS039686	ERP000746
CC23	RBH08	Australia	2008	Human	Carriage	Ia	ST23	*tet*(M) *erm*(TR)	G23-Tn*5801*	PE 2 x 75	ERS046923	ERP000746
CC23	B50VS	Australia	2008	Human	Carriage	Ia	ST24	*tet*(M)	G23-Tn*5801*	PE 2 x 75	ERS046929	ERP000746
CC23	B81VS	Australia	2008	Human	Carriage	Ia	ST23	*tet*(M)	G23-Tn*5801*	PE 2 x 75	ERS046931	ERP000746
CC23	B50VD	Australia	2008	Human	Carriage	Ia	ST23	*tet*(M)	G23-Tn*5801*	PE 2 x 75	ERS046910	ERP000746
CC23	Madagascar-IP-7	Madagascar	2006	Human	NA	Ia	ST23	*tet*(M)	G23-Tn*5801*	51	ERS337519	PRJEB4456
CC23	CCH210801006	Canada	2010	Human	Carriage	Ia	ST23	*tet*(M) *lsa*(C)	G23-Tn*5801*	110	ERS337511	PRJEB4456
CC23	CCH263	France	1998	Human	Urine	Ia	ST23	*tet*(M) *tet*(L)	G23-Tn*5801*	51	ERS337512	PRJEB4456
CC23	NEM1002	France	1996	Human	NInv-EOD	Ia	ST23	*tet*(M)	G23-Tn*5801*	51	ERS337521	PRJEB4456
CC23	Bangui-IP-6	C. A. F.$	2006	Human	NA	Ia	ST23	*tet*(M)	G23-Tn*5801*	51	ERS337510	PRJEB4456
CC23	BG-NI-001	Bulgaria	2009	Human	NCarriage	Ia	ST144-like#	*tet*(M)	G24-Tn*916*	PE 2 x 75	ERS039635	ERP000746
CC23	DK-NI-002	Denmark	2009	Human	NInv-EOD	V	ST23	*tet*(M)	G24-Tn*916*	PE 2 x 75	ERS039618	ERP000746
CC23	DE-NI-006	Germany	2008	Human	NInv-EOD	Ia	ST144-like#	*tet*(M)	G24-Tn*916*	PE 2 x 75	ERS039651	ERP000746
CC23	CZ-NI-001	Czech Republic	2008	Human	NCarriage	Ia	ST23-like#		G24-Tn*916*	PE 2 x 75	ERS039682	ERP000746
CC23	RBH07	Australia	2008	Human	Carriage	Ia	ST23	*tet*(M) *aadE aphA3 sta*	G24-Tn*916*	PE 2 x 75	ERS046922	ERP000746
CC23	928662	Germany	>2002	Animal	Dog	Ia	ST23	*tet*(M)	G24-Tn*916*	51	ERS337508	PRJEB4456
CC23	Madagascar-IP-79	Madagascar	2006	Human	NA	V	ST23	*tet*(M)	G24-Tn*916*	36	ERS337520	PRJEB4456
CC23	Madagascar-IP-11	Madagascar	2006	Human	NA	V	ST23	*tet*(M)	G24-Tn*916*	51	ERS337518	PRJEB4456
CC23	501_19	France		Animal	Bovine Mastitis	III	ST23	*tet*(M)	G25-Tn*5801*	51	ERS337509	PRJEB4456

Supplementary Table 1

CC23	CRBIP22.90	France	1953	Human	InvD	Ia	ST23	*tet*(M)	G26-Tn*5801*	51	ERS337516	PRJEB4456
CC23	DK-NI-016	Denmark	2010	Human	NCarriage	Ia	ST88-like[#]			PE 2 x 75	ERS039631	ERP000746
CC23	DE-NI-012	Germany	2009	Human	NInv-EOD	Ia	ST88-like[#]			PE 2 x 75	ERS039654	ERP000746
CC23	B42VD	Australia	2008	Human	Carriage	III	ST23-like[#]			PE 2 x 75	ERS046909	ERP000746
CC23	CRBIP22.110	France	1959	Human	Carriage	Ia	ST23			51	ERS337514	PRJEB4456
CC23	CCH350	France	2005	Human	AInvD	III	ST380			51	ERS337513	PRJEB4456
CC23	CRBIP22.97	France	1953	Human	InvD	Ia	ST23			51	ERS337517	PRJEB4456
CC23	CRBIP22.113	France	1959	Human	InvD	Ia	ST23			51	ERS337515	PRJEB4456
CC23	DK-NI-014	Denmark	2009	Human	NCarriage	Ia	ST88-like[#]	*tet*(O)		PE 2 x 75	ERS039629	ERP000746
CC26	Bangui-IP-105	C. A. F. [$]	2006	Human	Carriage	V	ST26	*tet*(M) *tet*(L)	G27-Tn*916*	51	ERS337537	PRJEB4456
CC26	Dakar-IP-30	Senegal	2006	Human	Carriage	V	ST26	*tet*(M)	G27-Tn*916*	36	ERS337539	PRJEB4456
CC26	Bangui-IP-84	C. A. F. [$]	2006	Human	Carriage	V	ST26	*tet*(M) *tet*(L)	G27-Tn*916*	51	ERS337538	PRJEB4456
CC26	IT-NI-036	Italy	2010	Human	NInv-EOD	V	ST26-like[#]	*tet*(M) *erm*(TR) *spcR*	G28-Tn*916*	PE 2 x 75	ERS039680	ERP000746
CC26	IT-NI-037	Italy	2010	Human	NInv-EOD	V	ST26-like[#]	*tet*(M) *erm*(TR) *spcR*	G28-Tn*916*	PE 2 x 75	ERS039681	ERP000746
CC26	39_07	Malaysia	1971	Bovine	Bovine Milk	V	ST26			51	ERS337536	PRJEB4456
	IT-NI-003	Italy	2008	Human	NInv-EOD	III	ST496-like[#]	*tet*(M)	Tn*5801*	PE 2 x 75	ERS039667	ERP000746
	Madagascar-IP-37	Madagascar	2007	Human	Carriage	Ia	ST314	*tet*(M)	Tn*5801*	110	ERS337545	PRJEB4456
	CCH298	France	2005	Human	Urine	Ia	ST248-like[#]	*tet*(M)	Tn*5801*	51	ERS337543	PRJEB4456
	GB-NI-014	Great Britain	2008	Human	NInv-EOD	II	ST22-like[#]	*tet*(M)	G29-Tn*916*	PE 2 x 75	ERS039706	ERP000746
	RBH06	Australia	2008	Human	Carriage	II	ST22	*tet*(M)	G29-Tn*916*	PE 2 x 75	ERS046921	ERP000746
	2584	France	1987	Bovine	Bovine mastitis	Ia	ST420-like[#-&]	*tet(M) lsa*(C)	Tn*916*	36	ERS337463	PRJEB4456
	Madagascar-IP-43	Madagascar	2007	Human	Carriage	Ia	ST103	*tet*(M)	Tn*916*	51	ERS337546	PRJEB4456
	IT-NI-016	Italy	2009	Human	NInv-EOD	IX	ST130-like[#]			PE 2 x 75	ERS039671	ERP000746
	GB-NI-002	Great Britain	2009	Human	NInv-EOD	IX	ST130-like[#]			PE 2 x 75	ERS039697	ERP000746

Supplementary Table 1

	ni1122	France	1985	Bovine	Bovine mastitis	II	ST418			36	ERS337505	PRJEB4456
	443_26	Germany	1971	Bovine	Bovine Mastitis	Ia	ST7			36	ERS337540	PRJEB4456
	Spain-IP-30	Spain	2005	Bovine	Bovine Milk	II	ST415-489like			51	ERS337548	PRJEB4456
	SS1219[1]	Israel	1994	Fish	NA	Ib	ST261					

[#] One of the seven alleles was in two contigs in the assembly; [$]Central African Republic; [&] Differs from the ST by a single SNP in one of the seven alleles; [§]NA: not availaible; InvD: invasive disease; NInv: neonatal invasive disease; EOD: early-onset disease; LOD: late onset disease; NCarriage: Neonatal carriage; AInv: adult invasive disease

1 Rosinski-Chupin I*, et al.* Reductive evolution in *Streptococcus agalactiae* and the emergence of a host adapted lineage. *BMC Genomics* **14**, 252 (2013).
